# Sensory Adaptation in the Whisker-Mediated Tactile System: Physiology, Theory, and Function

**DOI:** 10.3389/fnins.2021.770011

**Published:** 2021-10-29

**Authors:** Mehdi Adibi, Ilan Lampl

**Affiliations:** ^1^Department of Physiology and Biomedicine Discovery Institute, Monash University, Clayton, VIC, Australia; ^2^Department of Neuroscience and Padova Neuroscience Center (PNC), University of Padova, Padova, Italy; ^3^Department of Brain Sciences, Weizmann Institute of Science, Rehovot, Israel

**Keywords:** rodent, whisker system, somatosensory, neuronal adaptation, noise correlation, neural coding, information theory, neural network

## Abstract

In the natural environment, organisms are constantly exposed to a continuous stream of sensory input. The dynamics of sensory input changes with organism's behaviour and environmental context. The contextual variations may induce >100-fold change in the parameters of the stimulation that an animal experiences. Thus, it is vital for the organism to adapt to the new diet of stimulation. The response properties of neurons, in turn, dynamically adjust to the prevailing properties of sensory stimulation, a process known as “neuronal adaptation.” Neuronal adaptation is a ubiquitous phenomenon across all sensory modalities and occurs at different stages of processing from periphery to cortex. In spite of the wealth of research on contextual modulation and neuronal adaptation in visual and auditory systems, the neuronal and computational basis of sensory adaptation in somatosensory system is less understood. Here, we summarise the recent finding and views about the neuronal adaptation in the rodent whisker-mediated tactile system and further summarise the functional effect of neuronal adaptation on the response dynamics and encoding efficiency of neurons at single cell and population levels along the whisker-mediated touch system in rodents. Based on direct and indirect pieces of evidence presented here, we suggest sensory adaptation provides context-dependent functional mechanisms for noise reduction in sensory processing, salience processing and deviant stimulus detection, shift between integration and coincidence detection, band-pass frequency filtering, adjusting neuronal receptive fields, enhancing neural coding and improving discriminability around adapting stimuli, energy conservation, and disambiguating encoding of principal features of tactile stimuli.

## 1. Introduction

In the natural environment, organisms are constantly exposed to a continuous stream of sensory input. The dynamics of sensory input changes with organism's behaviour and environmental context. The contextual variations may induce over 100-fold change in the stimulation physical parameters describing the sensory environment that an animal experiences. Thus, it is vital for the organism to adapt to the new diet of stimulation. The response properties of neurons, in turn, dynamically adjust to the prevailing properties of sensory stimulation, a process known as “sensory adaptation.” Adaptation is a ubiquitous phenomenon across all sensory modalities and occurs at different stages of processing from periphery to cortex. The neuronal consequences of adaptation are conventionally characterised as suppression in responsiveness. However, the current view of adaptation is continuous retuning of neuronal response functions in the form of shifts or rescaling to compensate for the changes in the diet of stimulation (Fairhall et al., 2001; Dean et al., 2005; Sharpee et al., 2006; Adibi et al., 2013b). This adaptive recalibration is hypothesised to improve neural coding efficiency by changing neuronal response functions to match the statistics of the sensory environment (Barlow, 1961; Smirnakis et al., 1997; Kvale and Schreiner, 2004; Dean et al., 2005; Hosoya et al., 2005; Price et al., 2005; Nagel and Doupe, 2006; Maravall et al., 2007; Adibi et al., 2013a). The present article provides an analytic summary of current views and research findings within the last decade on sensory adaptation in the rodent whisker-mediated tactile system.

The rodent whisker system provides a suitable model system in systems neuroscience due to its functional efficiency and structural organisation (Brecht, 2007; Petersen, 2007; Feldmeyer, 2012; Feldmeyer et al., 2013; Adibi, 2019); known as nocturnal animals, rodents rely on their vibrissal sensorimotor system to garner information about their surrounding environment. Every stage of processing comprises anatomical and functional somato-topographic maps of whiskers: “barrelettes” in the brainstem nuclei, “barreloids” in the sensory thalamus ventral posteromedial nucleus (VPM), and “barrels” in the primary somatosensory cortex (S1). Behavioural studies demonstrated that rodents are able to perform texture discrimination (Carvell and Simons, 1990; von Heimendahl et al., 2007), vibration amplitude and frequency discrimination (Adibi and Arabzadeh, 2011; Morita et al., 2011; Adibi et al., 2012; Fassihi et al., 2014), object localisation (Mehta et al., 2007; O'Connor et al., 2010), gap crossing (Harris et al., 1999; Celikel and Sakmann, 2007) and aperture width discrimination (Krupa et al., 2001) tasks using their macro-vibrissae. In the whisker sensory pathway, sensory adaptation has been observed and quantified along various stages of sensory processing, from trigeminal ganglion through brainstem and sensory thalamic nuclei to somatosensory cortex. These quantifications are commonly in terms of a drop in neuronal responsiveness to sustained or repetitive whisker stimulation (Hartings et al., 2003; Khatri et al., 2004; Fraser et al., 2006; Ganmor et al., 2010; Adibi et al., 2013b; Mohar et al., 2013; Kheradpezhouh et al., 2017). However, adaptation operates in both ways. For instance, sensory adaptation enhances neuronal responses when the stimulation regime changes to a lower level of stimulation or lower adaptation. In this article, we focus on the fast/rapid neuronal adaptation to the immediate history of stimulus within the order of a few hundreds of milliseconds. We discuss key findings about the intensity-dependent properties of sensory adaptation and further summarise the effect of neuronal adaptation on response dynamics and encoding efficiency of neurons at single-cell and population levels along the whisker-mediated tactile system. Finally, we summarise the perceptual effects of adaptation, and its functional role from a cognitive and systems neuroscience point of view.

## 2. Phenomenology of Sensory Adaptation

**Figure 1:**
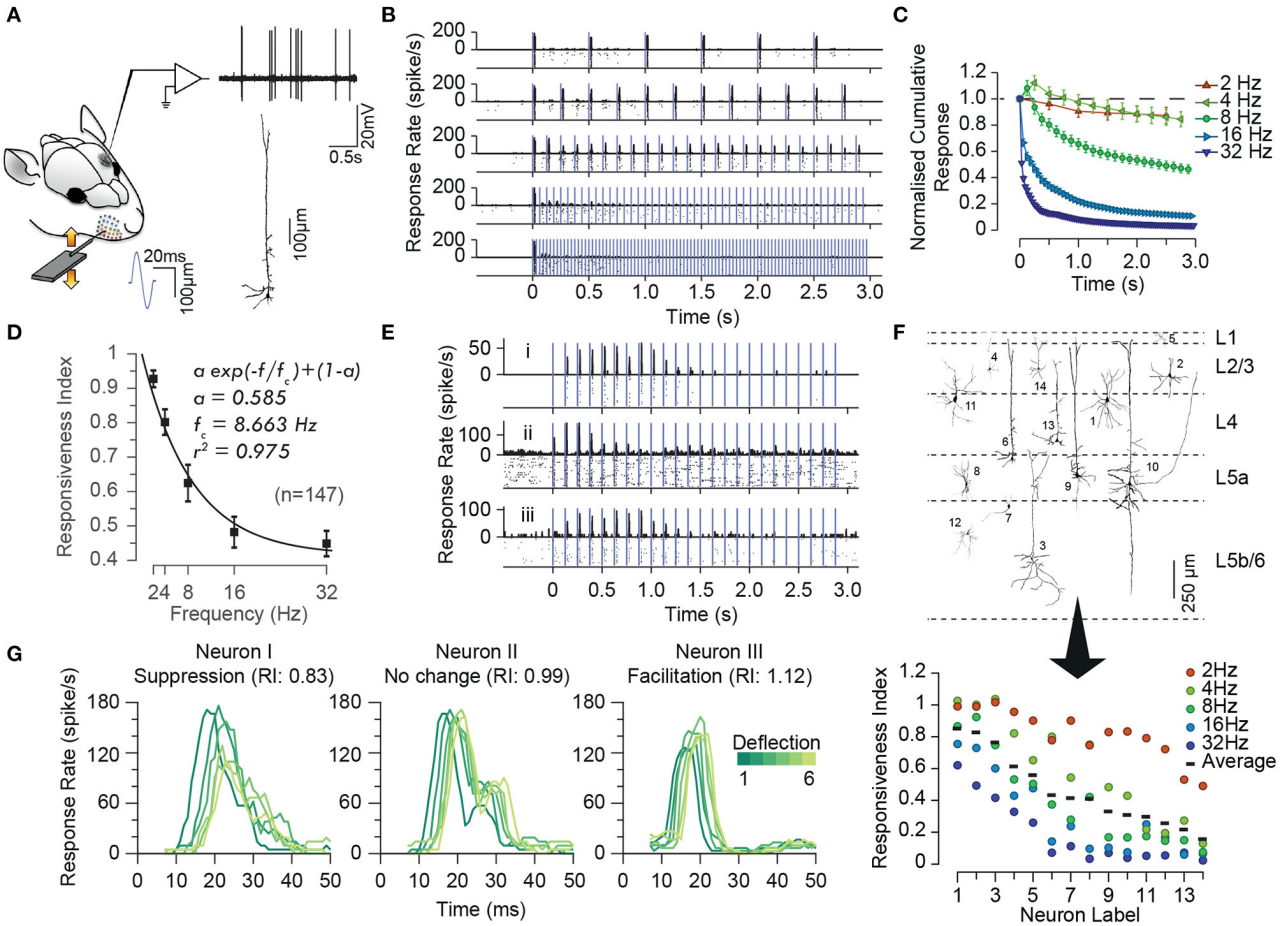
Characterisation of the adaptation profile to repetitive stimulation. **(A)** Histological reconstruction of a sample layer 5 pyramidal neuron juxta-cellularly recorded from the vibrissal area of S1 in anaesthetised rats while applying deflections (200 μm in amplitude) to the principal whisker. **(B)** Raster plots and peri-stimulus time histograms (PSTHs) for the sample neuron in **(A)** for different stimulation frequencies. Vertical purple lines indicate individual deflections. Dots in the lower parts of panels indicate individual spikes and rows correspond to trials. **(C)** The cumulative response of the sample neuron as a function of time normalised to the response to the first deflection exhibiting a systematic decrease in responsiveness with time. The decline is steeper and reaches a lower level as the stimulation frequency increased. Error bars indicate standard error of the means (s.e.m.) across trials. **(D)** On average, across neurons, adaptation increases with stimulation frequency in an exponential manner (solid curve). Responsiveness index (RI) is defined as the net neuronal response rate to the 3-s train of deflections divided by the response to the first deflection. Error bars indicate s.e.m. **(E)** Prominent response facilitation in a subset of neurons. The response profile of three sample neurons exhibiting response facilitation is shown at stimulation frequency of 8 Hz. **(F)** Neuronal reconstruction and diversity of adaptation. Upper panel illustrates the morphology of 14 example reconstructed neurons and their cortical location as identified by histology. The lower panel shows the diversity of adaptation for the 14 neurons. **(G)** Response latencies increases over the time course of stimulation, irrespective of the dynamics of their response rate (facilitation versus adaptation). PSTHs for 3 sample neurons to 6 consecutive deflections at 2 Hz stimulation. Different shades of green represent the order of deflection within the simulation train, with darker corresponding to earlier deflections. Modified from Kheradpezhouh et al. (2017).

**Figure 2:**
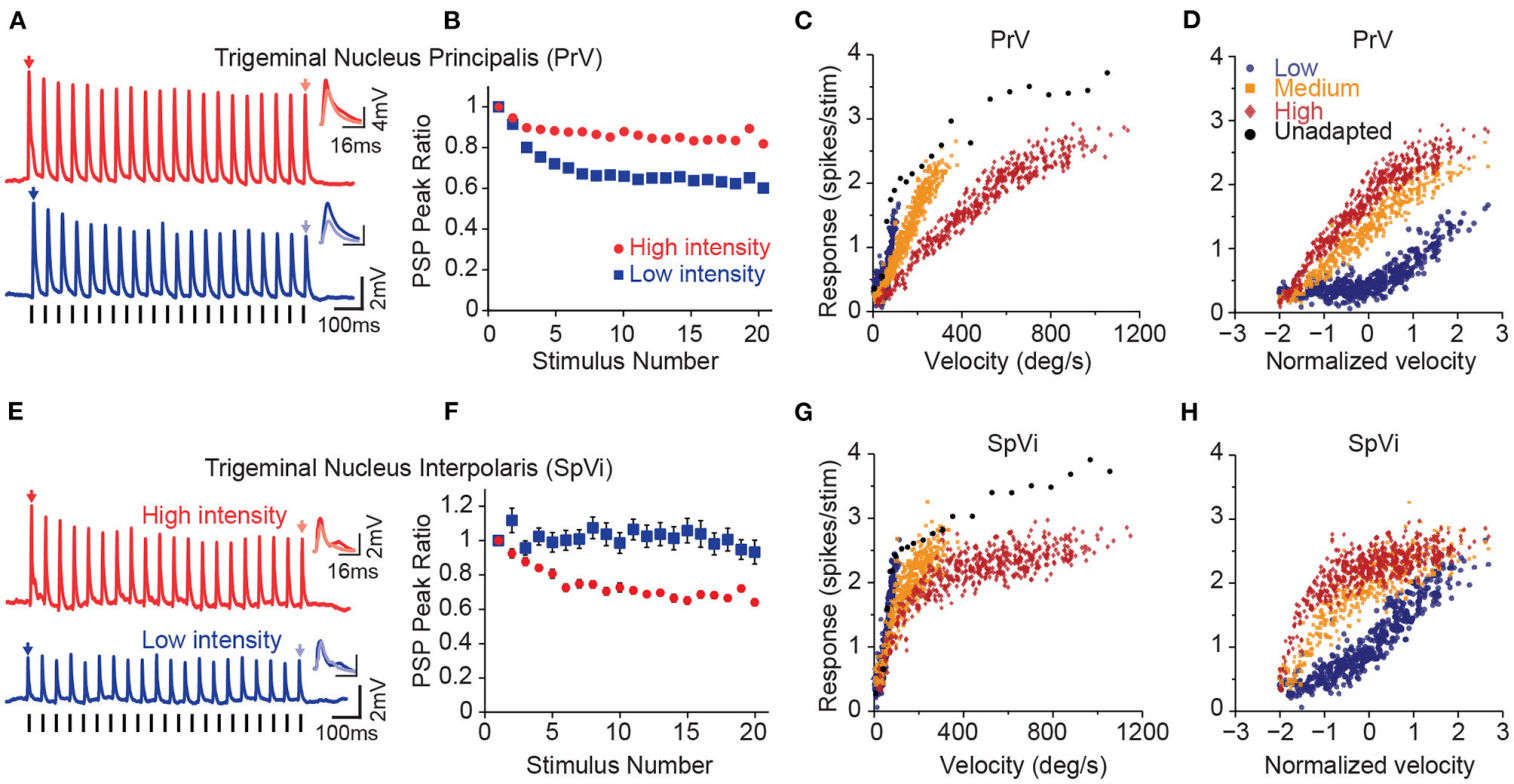
Parallel processing of tactile intensity during adaptation in the two trigeminal nuclei. **(A)** Sub-threshold post-synaptic responses of a PrV neuron to repeated low intensity input (blue) and high intensity stimulation (red). **(B)** Normalised peak sub-threshold response of PrV neurons for two intensities. PrV neurons exhibit less adaptation to the stronger stimulation. Error bars represent s.e.m. **(C)** Population averaged firing responses of PrV neurons as a function of stimulation velocity at low (blue circles), medium (orange squares), and high (red diamonds) intensity contexts. **(D)** Same data as in **(C)**, but re-plotted as a function of normalised (Z-scores) velocity. **(E)** Sub-threshold post-synaptic responses of a SpVi neuron to repeated high and low intensity stimulation. **(F)** Normalised peak sub-threshold response of PrV neurons for low and high intensities. In contrast to PrV, SpVi neurons adapt more to higher intensity stimulation. Error bars represent s.e.m. **(G)** Average firing response of SpVi neurons as a function of stimulation velocity. **(H)** as in **(G)**, but as a function of normalised (z-scored) velocity. Although unadapted responses (black circles) are similar for both nuclei, PrV encodes stimulus intensity more linearly at the high-intensity context and the SpVi better encodes the low-intensity context. Modified from Ganmor et al. (2010), Mohar et al. (2013), and Mohar et al. (2015).

**Figure 3:**
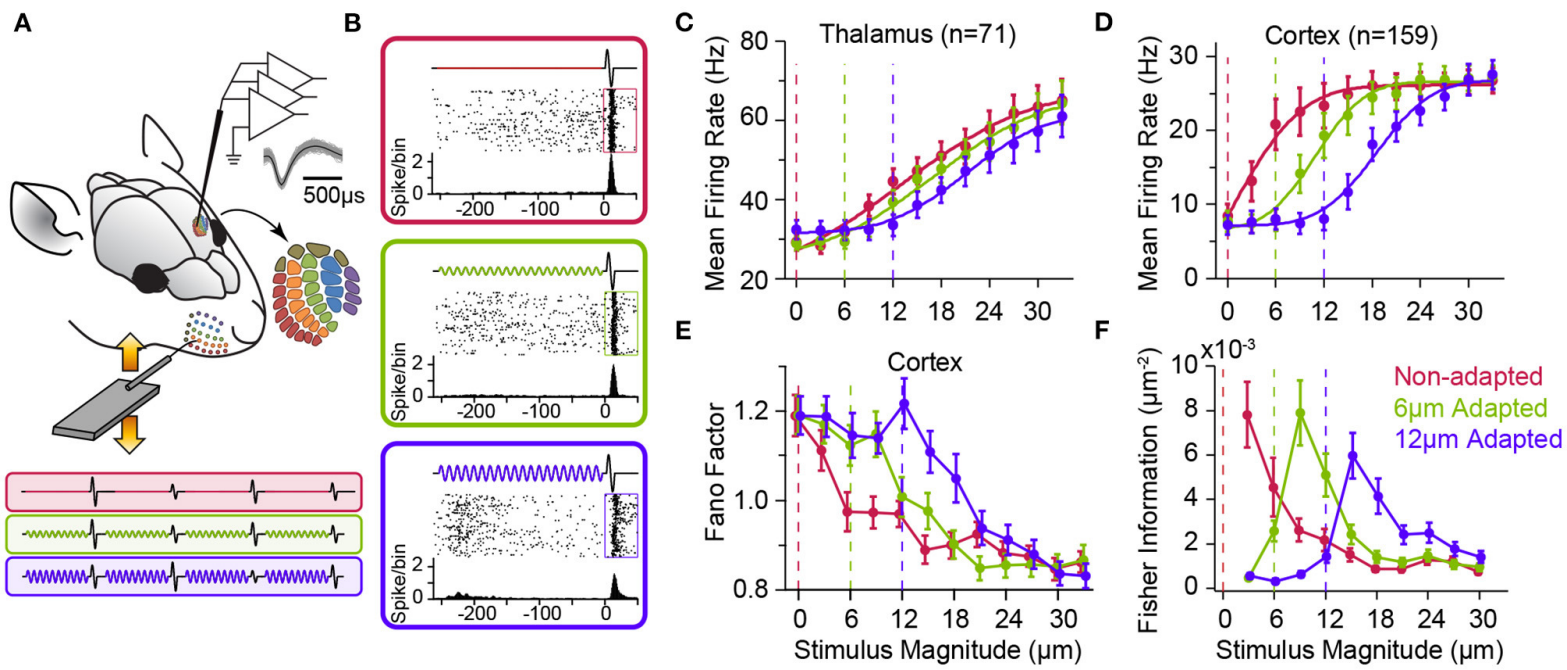
Adaptation to sustained whisker stimulation generates systematic shifts in neuronal response statistics. **(A)** Extracellular multi-electrode array recording in rat whisker area of S1. The inset depicts the somatotopic organisation of barrels in layer 4 and infra-barrels in layer 6a, and the isolated spikes of a typical cortical neuron recorded from Barrel D4 while stimulating the principal whisker under three adaptation conditions (red: 0, green: 6 and magenta: 12 μm, 250 ms long at 80 Hz) followed by a single-cycle sinusoidal whisker vibration (0–33 μm). **(B)** Each panel shows response of the sample neuron to the 30 μm test stimulus in each adaptation condition. PSTHs are calculated in a 5 ms long bin sliding in 1 ms steps over 100 trials. **(C)** Adaptation shifts the response function of thalamic neurons. Vertical dashed lines represent the magnitude of the adapting stimulus. **(D)** Adaptation generates systematic shifts in the average population response function of S1 neurons. The response of simultaneously recorded units were averaged together to produce a population spike rate for individual sessions (*n* = 8 comprising a total of 159 single- and multi-units). Spike rates are calculated in a 50 ms window after test stimulus onset (boxes in **B**). **(E)** Trial-to-trial variations in neuronal responses of cortical single units (*n* = 64) as captured by Fano factor (variance over mean spike counts across trials) exhibits a shift by adaptation. **(F)** The coding accuracy quantified by Fisher information peaks at amplitudes higher than that of the adapting stimulus. Single-neuron Fisher information as a function of stimulus magnitude (*n* = 73 single neurons). All error bars indicate s.e.m. Modified from Adibi et al. (2013a) and Adibi et al. (2013b).

As in any sensory modality, neurons in the somatosensory pathway exhibit adaptation to repeated or sustained whisker stimulation. The degree of adaptation depends on the stimulation parameters such as the frequency (Ahissar et al., 2000; Khatri et al., 2004; Heiss et al., 2008; Kheradpezhouh et al., 2017, see Figures 1A–D), the amplitude and velocity of whisker stimulation (Ganmor et al., 2010; Adibi et al., 2013b; Mohar et al., 2013, see Figures 2, 3), and the cortical and behavioural states (Castro-Alamancos, 2004a; Katz et al., 2012). The most recognised characteristic of neuronal adaptation is in the form of an exponential decrease in the neuronal responses to repeated sensory stimulation with time (Figures 1B,C) as well as with the frequency of stimulation (Figure 1D, see also Hartings et al., 2003; Khatri et al., 2004; Heiss et al., 2008; Kheradpezhouh et al., 2017). However, juxta-cellular electrophysiology and labelling of neurons in the primary somatosensory cortex (Kheradpezhouh et al., 2017) revealed the diversity of adaptation profiles including facilitation and increased evoked responses over time (Figure 1E, also see below) and with the frequency of stimulation. This diversity was not found to be correlated with the morphology of cortical neurons or their location across cortical laminae (Figure 1F, also see Musall et al., 2014; Ramirez et al., 2014; Allitt et al., 2017). Interestingly, a ubiquitous effect of sensory adaptation is increased response latency with respect to the onset of each consecutive whisker stimulation (Figure 1G).

### 2.1. Sensory Adaptation Along the Pathway: From Periphery to Cortex

In the rodent whisker-mediated tactile system, neuronal adaptation is observed across all stages of sensory processing, from the whisker follicle through the brainstem and the thalamus to the somatosensory cortex. Vibrissae, or whiskers are the starting point of this system. The instantaneous velocity of whisker movement is one of the fundamental kinematic features of whisker-mediated sensation in both modes of sensation, the receptive (passive) mode and during whisking (active or generative mode). Whisker velocity has been shown to be related to the radial distance of stationary and moving objects during contact with whiskers (Bagdasarian et al., 2013; Lottem et al., 2015) as well as the speed of the moving object (Lottem et al., 2015). Additionally, profile of whisker velocity determines texture-specific kinetic signatures through sequence of stick-slip events—discrete high-velocity, high-acceleration whisker micro-motions—during contact with objects (Arabzadeh et al., 2005). As such, the body of the literature commonly characterises tactile stimuli in terms of the velocity of whisker movement.

The first stage of sensory processing, the trigeminal ganglion (also known as semilunar ganglion), consists of the cell bodies of pseudo-unipolar neurons with their distal axons arborising the vibrissae follicles and shaft of the mystacial whiskers as mechanosensory receptors (Vincent, 1913; Ma and Woolsey, 1984, for a review see Adibi, 2019). The proximal axons of trigeminal primary sensory neurons innervate the ipsilateral brainstem trigeminal complex (Vincent, 1913; Ma and Woolsey, 1984). Each ganglion cell innervates only one whisker follicle (Fitzgerald, 1940; Zucker and Welker, 1969; Dykes, 1975; Gibson and Welker, 1983; Rice et al., 1986; Lichtenstein et al., 1990). Each follicle is innervated by 150–200 myelinated and around 100 unmyelinated distal axons of trigeminal ganglion neurons (Lee and Woolsey, 1975; Waite and Cragg, 1982; Renehan and Munger, 1986; Rice et al., 1986, 1997; Henderson and Jacquin, 1995). The nerve terminals and mechanoreceptors are of various types, morphologies and distributions (Melaragno and Montagna, 1953) such as Merkel cell-neurite complexes, lanceolate receptors, club-like endings, Ruffini-like corpuscles—also referred to as reticular ending—and free nerve endings (Renehan and Munger, 1986; Rice et al., 1986; Ebara et al., 2002). Early studies classified the trigeminal ganglion neurons into slowly adapting and rapidly adapting based on their response profile within the first milliseconds to a rapid change in whisker angle. Rapidly adapting receptors do not elicit response to maintained whisker deflection, while slowly adapting receptors respond to sustained whisker deflection (Talbot et al., 1968; Kwegyir-Afful et al., 2008). The relative time interval between velocity-independent first spike latency of rapidly adapting neurons and velocity-dependent first spike latency of slowly adapting neurons accurately and reliably encodes whisker movement velocity (Lottem et al., 2015). Whisker velocity is also encoded, although less robustly, by the firing rates of slowly adapting neurons (Shoykhet et al., 2000; Bale et al., 2013; Lottem et al., 2015). Merkel endings are the most prominent mechanoreceptors with slowly adapting characteristics (type I skin Merkel endings in mouse trunk and ring-sinus Merkel receptors in rat vibrissal follicles) (Iggo and Muir, 1969; Woodbury and Koerber, 2007; Furuta et al., 2020). Club-like, rete-ridge collar Merkel and lanceolate receptors are rapidly adapting while reticular-like type-I Ruffini endings exhibit slowly adapting characteristics (Li and Ginty, 2014; Tonomura et al., 2015; Furuta et al., 2020). Rapidly adapting ganglion cells have generally higher velocity thresholds (Zucker and Welker, 1969; Lichtenstein et al., 1990). The time course of adaptation in trigeminal ganglion neurons and mechanoreceptors is in the order of a few tens of milliseconds. In contrast to cortical (Khatri et al., 2004; Musall et al., 2014; Allitt et al., 2017; Kheradpezhouh et al., 2017, see Figure 1), thalamic (Hartings et al., 2003; Khatri et al., 2004; Ganmor et al., 2010) and brainstem neurons (Mohar et al., 2013), the response of trigeminal ganglion neurons to repeated deflections at stimulation frequencies as high as 18 Hz exhibits little adaptation (Ganmor et al., 2010).

Proximal axons of the first-order trigeminal ganglion neurons innervate the two sensory nuclei in ipsilateral brainstem trigeminal complex: the principal sensory nucleus (PrV) and the spinal nucleus (SpV). The PrV and SpV interpolaris sub-nucleus (SpVi) provide the main sensory input to the thalamus forming the starting point of the two major parallel streams of somatosensory signals: the lemniscal and the paralemniscal pathways (Yu et al., 2006, for a review see Adibi, 2019). Neurons in these two sub-nuclei exhibit opposite intensity-dependent adaptation profiles to repeated deflections of the principal whisker (Figure 2); PrV neurons adapt less to higher intensity stimuli (Figures 2A,B), while neurons in SpVi exhibit increased adaptation as the intensity of deflections increases (Mohar et al., 2013, see Figures 2E,F). The intensity-dependent adaptation feature in PrV neurons is preserved at the level of VPM and cortical neurons (Ganmor et al., 2010; Mohar et al., 2013, 2015). While the neuronal mechanisms of intensity-dependent adaptation in SpVi and PrV neurons remain unknown, these findings suggest that neuronal adaptation in PrV may be due to the inter-subnuclear inhibition of PrV neurons by the SpVi (Furuta et al., 2008); as the intensity of stimulus increases, SpVi neurons adapt more causing a greater disinhibition of the PrV neurons. The disinhibition of PrV neurons to repeated whisker deflections, in turn, decreases the level of adaptation at high-intensity stimulation regime; PrV neurons adapt less to higher intensity stimuli, while neurons in SpVi exhibit increased adaptation as the intensity of deflections increases (Mohar et al., 2013). The intensity-dependent pattern of adaptation in PrV neurons further is preserved at the level of VPM and cortical neurons (Ganmor et al., 2010; Mohar et al., 2013, 2015). Increasing the amplitude and velocity of whisker deflections does not increase the adaptation of synaptic responses in layer 4 neurons of the somatosensory cortex, but rather entails less adaptation (Ganmor et al., 2010). Importantly, previous studies (Timofeeva et al., 2004) suggested that inter-subnuclear interactions between SpVi inputs shape the receptive field size of PrV neurons. Indeed, the pattern of intensity-dependent adaptation in PrV neurons is reversed when the adjacent whisker is stimulated. That is, increasing the intensity of stimulation entails less adaptation when stimulation intensity increases.

Although the two trigeminal nuclei encode the intensity of whisker stimuli in a similar manner under non-adapted condition (Figures 2C,G), adaptation introduces distinct changes in the coding behaviour of these two nuclei (Mohar et al., 2015). Under adaptation, PrV neurons better encode the fluctuations of the stimulus at high intensity regimes (Figures 2C,D), whereas SpVi neurons better encode weak tactile stimuli (Figures 2G,H). A similar pattern was also observed at the level of the subthreshold synaptic potentials (Mohar et al., 2015). As the neuronal adaptation linearises the response function of PrV neurons at high-intensity stimulation regime, it linearises the response function of SpVi neurons at low-intensity stimulation regime. Thus, the two parallel routes of stimulus intensity processing through PrV and SpVi together enhance the overall coding of stimulus intensity for a broader stimulation range. These findings suggest that neurons belonging to these two brainstem trigeminal nuclei may encode the intensity of stimulation together in a context-dependent manner untangling the coding ambiguity associated with response adaptation in different stimulation contexts.

Along the pathway from periphery to cortex, adaptation exhibits stronger effect on neuronal responses. Peripheral trigeminal ganglion neurons exhibit less adaptation than neurons in the principal nucleus of the brainstem trigeminal complex (PrV). Sensory thalamic neurons in the VPM, in turn, exhibit a higher level of adaptation than neurons in trigeminal complex, and less adaptation compared to cortical neurons (Khatri et al., 2004; Ganmor et al., 2010, also see Figures 3C,D). As mentioned earlier, the degree of adaptation depends on the frequency of whisker stimulation (Ahissar et al., 2000; Khatri et al., 2004; Heiss et al., 2008; Kheradpezhouh et al., 2017; Latimer et al., 2019), the amplitude and velocity of deflections (Ganmor et al., 2010; Adibi et al., 2013b; Mohar et al., 2013), and the cortical state (Castro-Alamancos, 2004a; Katz et al., 2012). The response of the neurons decreases to consecutive individual whisker deflections with an exponential decay (Figure 1C). As the stimulation frequency increases, neurons adapt stronger and at a faster rate (Khatri et al., 2004; Kheradpezhouh et al., 2017, also see Figure 1D). A significant subset of cortical neurons exhibit response facilitation over the course of repetitive stimulation at frequencies of 4–10 Hz (Brecht and Sakmann, 2002; Garabedian et al., 2003; Derdikman et al., 2006; Kheradpezhouh et al., 2017, see Figure 1E). The facilitation is then followed by a reduced responsiveness to further subsequent deflections (Kheradpezhouh et al., 2017). Similar facilitation effects are reported in response to repetitive optogenetic excitation of layer 6 corticothalamic neurons where the evoked response in layer 5a pyramidal neurons as well as fast-spiking inter-neurons in both layer 4 and 5a increased due to activation of facilitating synapses (Kim et al., 2014). Prolonged optogenetic activation of layer 6 corticothalamic neurons resulted in a hyper-polarisation of VPM neurons followed by depolarisation, shifting the mode of sensory responses from bursting to single-spike (Mease et al., 2014). Subsequently, VPM neurons exhibit reduced adaptation to 8 Hz repetitive whisker deflections during prolonged optogenetic stimulation of layer 6 neurons. This suggests corticothalamic feedback shapes both the gain and the temporal profile of sensory processing in cortex by controlling the gating of sensory information in VPM. Post-adaptation response facilitation was observed to stimulation at a few hundred milliseconds after the adapting repetitive stimulation in approximately a third of cortical neurons (Malina et al., 2013). Thalamic neurons, on the other hand, do not exhibit post-adaptation response facilitation revealing that facilitation does not emerge from thalamic neurons. Recordings at different holding currents revealed this facilitation is a result of a faster recovery of excitation compared to inhibition. Adaptation also is shown to decrease cross-whisker suppression, similar to the reduction in surround suppression in visual system (Higley and Contreras, 2006; Ramirez et al., 2014).

Early electrophysiology studies revealed that on average, neuronal responses from layers 2/3 and 5a exhibit stronger adaptation than cortical layers 4 and 5b (main lemniscal input layers) neurons (Ahissar et al., 2001; Derdikman et al., 2006). These findings are consistent with stronger adaptation in the posterior medial (POm) thalamus than in VPM (Diamond et al., 1992; Sosnik et al., 2001). In a detailed study of adaptation to sequences of stick-slip events across cortical laminae, Allitt et al. (2017) found stronger adaptation in supra-granular layers compared to layers 4 and 5. Lower layer 3 showed rates of adaptation that lie between that of layers 2/upper 3 and layers 4/5. Despite strong responses to high-speed protraction (at 654°/s) resulting in suppressed responses to the initial stick-slip event in the sequence, across all laminae, the rate of adaptation vs. frequency did not change with the speed of protraction (654°/s vs. 32.7°/s). However, layer 2 neurons represent initial texture-defining stick-slip events with temporal fidelity and relatively high firing rates irrespective of protraction speed, and only exhibit adaptation to the subsequent repetitive stick-slip stimuli, and not to the strong evoked responses by fast whisker protraction. Interestingly, stick-slip stimuli in Allitt et al. (2017) drive weaker rates of adaptation at stimulation frequencies as high as 34 Hz compared to pulsatile whisker deflections at similar range of frequencies which disrupt texture encoding reported in previous studies (Ahissar et al., 2001; Chung et al., 2002; Khatri et al., 2004).

In the somatosensory cortex, both response latency and response peak time (to repeated whisker deflections) show a consistent increase over time with consecutive stimulations, irrespective of the direction and amount of the change in response rate (facilitation and suppression, as shown in Figure 1G, Allitt et al., 2017; Kheradpezhouh et al., 2017). This delayed response trend was also observed for neurons exhibiting little adaptation as well as those neurons exhibiting either decreased or increased responsiveness with repetitive stimulation. At stimulation frequencies >30 Hz, a large increase in latency from the 1st to 2nd stimulus was observed, followed by a decrease in response latency to a relative steady-state latency longer than that to the 1st stimulus and shorter than that for the 2nd stimulus (Allitt et al., 2017). These findings indicate that the context-dependent changes in the evoked response latency is governed by additional mechanisms than those underlying decrease or increase in the responsiveness.

### 2.2. Effect of Adaptation on Neuronal Response Characteristic Functions

As shown in Figure 2, adaptation exhibits differential effects depending on the intensity, frequency and potentially other physical parameters of sensory stimulation (also see Katz et al., 2006; Ganmor et al., 2010; Lampl and Katz, 2017; Katz and Lampl, 2021). Thus, it is important to characterise adaptation effects across a range of stimulus intensities. Using multi-electrode extracellular electrophysiology in anaesthetised rats (Figure 3A), Adibi et al. (2013b) quantified how neuronal adaptation modifies the input-output response function of neurons as a function of the whisker deflection intensity (amplitude). The neuronal input-output functions (also known as neurometric functions) typically exhibit an increase in the mean neuronal responses with stimulus amplitude (Figures 3B–D), along with decreased trial-by-trial variability (in terms of Fano factor, Figure 3E). Adibi et al. (2013b) delivered sustained sinusoidal vibrations at various amplitudes to whiskers to induce different levels of sensory adaptation, and then quantified the neuronal input-output function under each level of adaptation (Figures 3A,B). The findings revealed adaptation induces a rightward shift in the neuronal characteristic response functions (both the mean and variability against stimulus amplitude, Figure 3). The magnitude of the shift in neuronal responses depends on the magnitude of adapting stimulus; adaptation shifted the threshold of neuronal responses (the lowest stimulus intensity to which the evoked response is significantly higher than baseline activity) to stimulation amplitudes above that of adapting stimulus (Figures 3C,D). While adaptation shifts the neuronal characteristic functions (response rate and variability), it maintains the relationship between the two across different adaptation states. This was confirmed by a regression analysis between the response variability and mean (Adibi et al., 2013b). Thus, adaptation transfers the operating point of neurons to lower rates with higher variability. It is worth to note that the lateral shift in response function lowers overall responsiveness (spike counts averaged across the whole stimulus range) which in turn, suggests a lower metabolic cost. Consequently, the coding accuracy (in terms of Fisher Information) peaks at amplitudes above the adapting stimulus (Figure 3F, see also Adibi et al., 2013a). Sensory adaptation produces systematic rightward shifts in the stimulus region with elevated coding efficiency consistent with the shift in the evoked neuronal response thresholds to amplitudes higher than that of the adapting stimulus (Adibi et al., 2013b).

### 2.3. Effect of Adaptation on the Network: Signal and Noise Correlations

**Figure 4:**
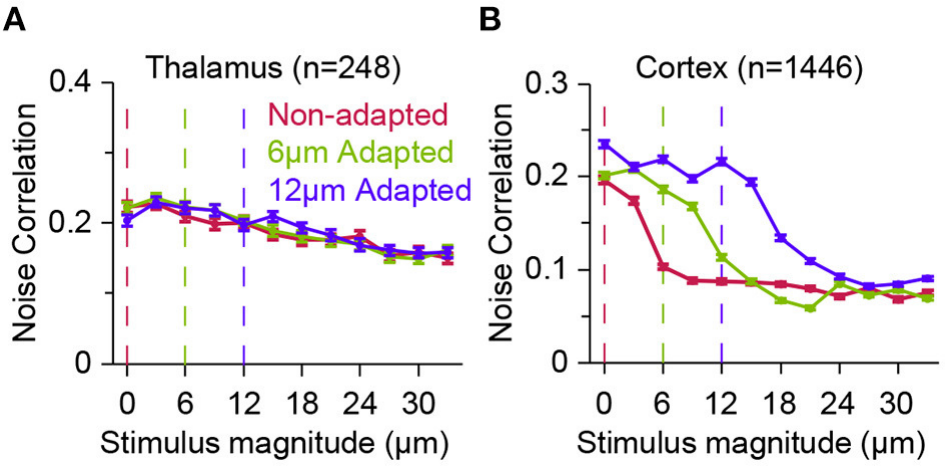
Effect of adaptation on population response correlations along the thalamocortical pathway. **(A)** The correlations in trial-by-trial spike-count variability (known as noise correlation) for a population of thalamic neurons in three adaptation states, as in Figure 3. Adaptation has small effect on the correlated variability across thalamic neurons. Error bars represent s.e.m. Colour conventions as in Figure 3. **(B)** Similar to **(A)** but for cortical neurons. Adaptation produces a systematic shift in the noise correlation characteristic function. Modified from Adibi et al. (2013b).

Shared neural variability is a ubiquitous phenomenon in neural networks. Conventionally, neuronal activity has been characterised by the average and variance of responses over multiple trials. However, single neuron statistics do not capture the stochastic and dynamic characteristics of a neural network. Correlated fluctuations across neurons—known as noise correlations—are one of the central bases of the recent theories of neural computation (Pouget et al., 2013). These correlations are shown to affect the information content of population activity in the cortex (Averbeck et al., 2006; Adibi et al., 2013a,b). Previous electrophysiology studies indicated sensory stimulation decorrelates the population responses in somatosensory cortex (Middleton et al., 2012; Adibi et al., 2013b). Similar stimulus-driven decorrelation was observed in the primary visual cortex of primates (Kohn and Smith, 2005), middle temporal (MT) cortex (Ponce-Alvarez et al., 2013) and anterior superior temporal sulcus of macaque monkeys (Oram, 2011). Sensory adaptation shifts the profile of noise correlations in cortex along the stimulus amplitude axis similar to the shift in the other response statistics including mean and variability of responses (Figures 3, 4). These parallel shifts result in maintaining the relationship between noise correlations and the mean firing rate across different states of adaptation (Adibi et al., 2013b). Thus, the net effect of sensory adaptation in the cortex is to decrease the overall neuronal response rate across the stimuli while increasing the total variability as well as correlations in variability (noise correlation) across neurons in the cortex.

In contrast, at the upstream sensory stage to somatosensory cortex, in VPM, while thalamic neurons exhibit adaptation in form of a shift in the neurometric curves (Figure 3C), the noise correlation does not show visible difference across stimuli and adaptation states (Figure 4A). This was the case, even though the firing rate of thalamic neurons, on average, increased over two-fold from about 30 Hz at spontaneous level to 60 Hz with increasing the stimulus amplitude. In contrast, on average, a less than 25 Hz change in the response of cortical neurons to stimulation was accompanied by a halved level of noise correlations in the somatosensory cortex (Figure 4B).

**Figure 5:**
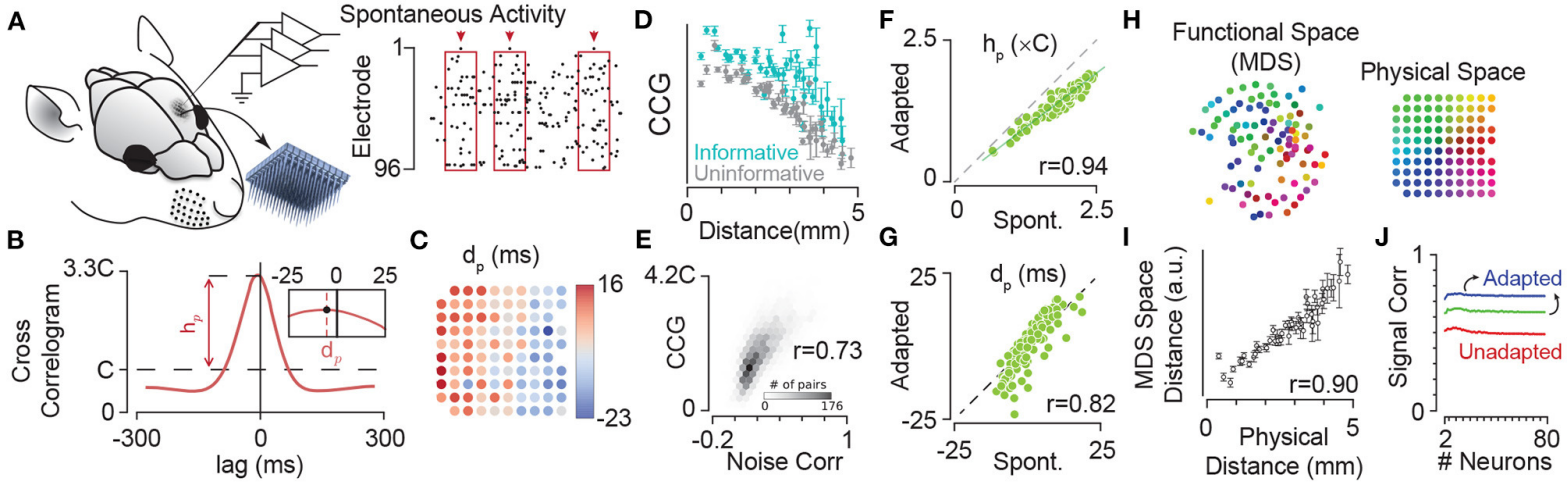
Adaptation maintains dynamics of synchrony in cortical neuronal populations. **(A)** Extracellular multi-electrode array recording in rat somatosensory cortex using a 10 × 10 array. The inset depicts the 10 × 10 array with 400 μm electrode spacing. **(B)** The probability density function (PDF) of the population activity triggered by spikes on an example electrode (arrows in **A**). This is identical to the cross-correlogram analysis. The peak of this PDF relative to chance level (denoted by **C**), represented by h_p_, quantifies the synchrony between each electrode and the pooled activity. The inset depicts the median of the PDF, denoted by d_p_, which estimates the delay (or lead) of each electrode relative to the population spiking at all other electrodes. **(C)** Map of electrodes colour coded by their corresponding d_p_ values. The d_p_ changes systematically from positive (leading the population) to negative (lagged relative to the population) most evident along the rows corresponded to the medio-lateral stereotaxic axis. **(D)** The mean and s.e.m. of strength of correlations (CCG) in spontaneous activity for informative pairs of units (where both electrodes in a pair were informative about sensory stimuli; cyan) and uninformative pairs (where both electrodes had low information about sensory stimuli; grey). Pairwise CCGs were calculated similar to that in **(B)**, but across two electrodes. **(E)** The histogram of correlations in spontaneous activity (normalised to the chance level C) against spike-count correlations in the fluctuations of evoked neuronal activity (noise correlations) showing significant strong relation between them. **(F)** Adaptation maintains the structure of correlations across the network. Strength of coupling in the spontaneous activity (non-adapted state, the abscissa) is highly correlated (correlation coefficient, *r* = 0.94) with those during sustained sensory stimulation (adapted condition, the ordinate). Each circle corresponds to an electrode. **(G)** Same as in **(F)**, but for d_p_. The values of d_p_ for episodes of spontaneous activity were highly correlated with those for the sustained stimulation (*r* = 0.82). This indicated that the sequence of activation among electrodes is highly preserved across the spontaneous (non-adapted) and adapted conditions. **(H)** The position of electrodes in the functional space built based on the pairwise CCG values from **(D)**. The functional space is reduced to two dimensions with multi-dimensional scaling (MDS). Colours of electrodes were assigned based on their spatial position as shown in the inset array. The spatial structure of coupling across neuronal population predicts the physical position of electrodes. **(I)** The mean and s.e.m. of distances in the 2-dimensional functional space at each physical distance. For dimensions higher than two, changes in the relation of functional space and anatomical space remained relatively small (less than 5%). **(J)** Adaptation increases signal correlations—correlations across response functions across stimuli—in neuronal populations. Signal correlation as a function of population size under the three adaptation states as in Figure 3. Colour convention is identical to Figure 3. Modified from Adibi et al. (2014) and Sabri et al. (2016).

By extracellular electrophysiology using a 10 × 10 electrode array from the somatosensory cortex (Figures 5A,B), Sabri et al. (2016) showed that neurons have a specific sequence of activation with respect to the population which is anatomically organised (Figure 5C). Additionally, the strength of pairwise correlations in the ongoing spontaneous activity of neuronal clusters decreases with the distance between the electrodes (Figure 5D), consistent with similar findings in other cortical areas (Smith and Kohn, 2008; Rothschild et al., 2010; Solomon et al., 2015). Correlations, on average, are stronger for units that better encode the sensory stimuli (Figure 5D), and predict the correlations in the evoked response fluctuations to sensory stimuli (i.e., noise correlations, Figure 5E) as well as signal correlations (see Sabri et al., 2016). The strength of correlations in spontaneous state (i.e., non-adapted) and adapted state (during sustained sensory stimulation) were highly correlated (Figures 5F,G, also see Sabri et al., 2016), indicating that neuronal adaptation maintains the spatiotemporal dynamics of population activity within the cortical networks; the functional connectivity map based on these correlations resembles the two-dimensional anatomical organisation of electrode locations (Figures 5H,I), and maintains its organisation across states of adaptation. Similarly, our unpublished data indicates neuronal adaptation maintains the spatial organisation of synchrony in the cortex as captured by the phase coupling of field potential oscillations (data not shown).

The shifts in the neuronal response functions by sensory adaptation cause an increased signal correlation (Figure 5J); sensory adaptation reduces the level of network heterogeneity by shifting the response function of neurons, aligning their responsive range with respect to the adaptor. Similar homeostatic effect of adaptation has been reported in the primary visual cortex of anaesthetised cats (Benucci et al., 2013). Benucci et al. (2013) observed that adaptation to a given orientation maintained the equality in responsiveness and the independence in orientation selectivity across the population.

### 2.4. Adaptation and Readout of Population Activity

**Figure 6:**
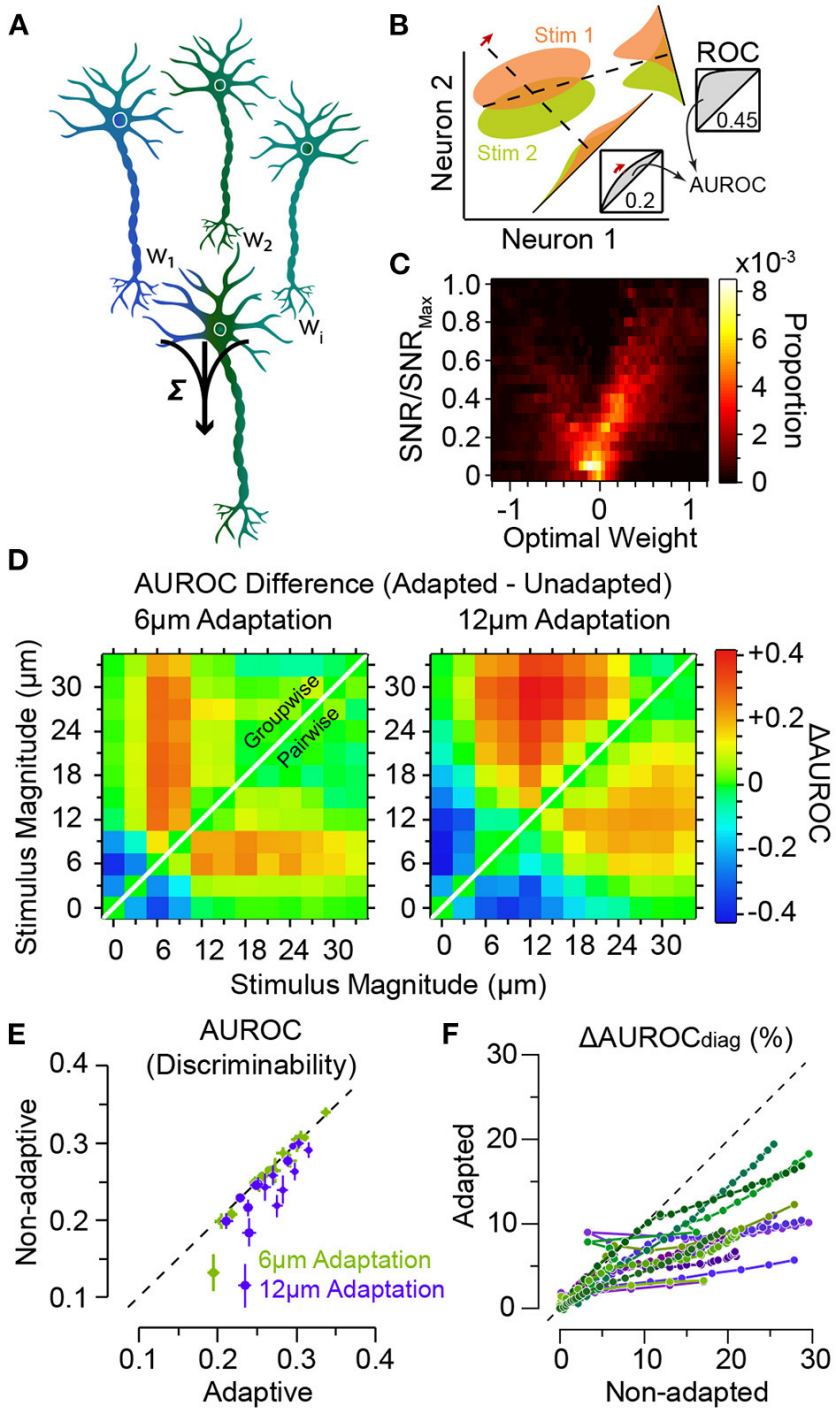
Adaptation improves population decoding. **(A)** Schematic representation of linear combination of neuronal activity by the downstream decoder. Coefficients *w*_1_, *w*_2_ and *w*_*i*_ represent the synaptic weights between the neurons (top row) and the decoder (bottom). **(B)** Schematic representation of pooling (summation along identity line) and optimal decoding. The green and orange ovals represent the joint distribution of the neurons' responses to two sensory stimuli. The solid black lines represent the weight vectors. The weight vector corresponding to pooling is along the identity line. The bell-shaped areas on each weight vector represent the projection of the neuronal response distribution for each stimulus on the weight vector. Dashed lines correspond to the best criterion to discriminate the two stimuli. The insets show the hit rate versus false alarm rate (ROC) for every possible criterion. Grey shaded areas indicate area under ROC (AUROC) quantifying discriminability. **(C)** The optimal decoding weights for informative neurons are higher. Histogram of the optimal weights as a function of the signal-to-noise ratio (SNR) for each stimulus pair across populations of 8 single neurons. The weights and SNR values are normalised to that of the best neuron in each population. **(D)** Shift in discriminability from low amplitudes to amplitudes higher than the adapting stimulus for every stimulus pair, in a sample session with 11 simultaneously recorded single neurons. Left and right panels exhibit the difference in discriminability (in terms of AUROC) between adapted and non-adapted conditions. Pairwise decoding applies a distinct set of weights for every stimulus pair, while the groupwise decoding applies an identical weight vector to discriminate across all stimulus pairs. **(E)** Decoding generalisation across adaptation states. The abscissa indicates the discriminability for the adaptive optimal decoder when optimised on half of the adapted responses and tested on the other half. The ordinate corresponds to discriminability for the non-adaptive optimal decoder when optimised on the non-adapted responses and tested on the adapted responses. Error bars indicate s.e.m. **(F)** The per cent drop in discriminability when ignoring noise correlations, denoted by ΔAUROC_diag_, for adaptation states, against the same measure in the non-adapted state. While noise correlations are higher in adapted states, the effect of ignoring these noise correlations under adaptation states is less compared to non-adapted state. Modified from Adibi et al. (2014).

How do the adaptation-induced shifts in characteristic functions (mean, variability and noise correlations) affect the efficiency of *readout* mechanisms of neuronal activity in downstream areas? A biologically plausible and efficient yet simple readout mechanism of the neuronal responses is a linear combination of the neuronal responses in a downstream neuron (decoder, see Figure 6A). The coefficients of the linear combination identify the synaptic weights between the neurons, and may be optimised to maximise the flow of sensory information or the decoder's discrimination performance (Figure 6B). The optimum weights depend on the amount of information each individual upstream neuron carries about the sensory stimuli (Figure 6C) and the structure of response co-variabilities across the population of downstream neurons (Adibi et al., 2014). This optimal linear readout determines an upper boundary of coding efficiency using the linear integration framework. Adibi et al. (2014) optimised the readout in two manners: (i) the pairwise-optimal readout scheme where for any pair of stimuli, the linear combination weights were optimised to maximise discriminability. And (ii) the groupwise-optimal readout scheme where an identical set of weights were optimised to maximise the discrimination across all stimuli. The discriminability of neuronal responses under pairwise-optimal readout provides an upper bound for the performance of the groupwise-optimal readout. The groupwise-optimal readout approaches its upper bound when the neuronal responses to sensory stimuli are linearly correlated. This is equivalent to a maximal level of signal correlation in the population responses. In order to apply the appropriate set of weights, the pairwise-optimal readout scheme requires *a priori* knowledge about the pair of stimuli to be discriminated. Thus, the groupwise-optimal readout is arguably a more biologically plausible scheme.

Adibi et al. (2014) found that for either readout scheme, adaptation enhances the discriminability for stimuli higher in amplitude than the adaptor, while there is a decline in discriminability if both stimuli are lower than the adaptor (Figure 6D). The magnitude of the effect was larger for the groupwise-optimal readout compared to the pairwise-optimal readout. These findings represent a shift in discriminability from low amplitudes to amplitudes higher than the adapting stimulus, consistent with the observed rightward shift in the neuronal response functions in Figure 3D. Additionally, adaptation increases the number of stimulus pairs with enhanced discriminability based on population responses (shades of red in Figure 6D).

As a result of the shifts in neuronal responses (Figure 3), the optimal readout weights which maximise discriminability between a pair of stimuli in the non-adapted state are expected to maximise discriminability between a new pair of stimuli that are in effect simply shifted by the adapting stimulus intensity. This predicts a high level of generalisation of the optimal readout across different states of adaptation. Adibi et al. (2014) verified this by quantifying the discriminability obtained from a non-adaptive readout—which its weights were optimised in the non-adapted state—relative to an adaptive readout—which its weights were optimised under the adaptation state. The results revealed that on average, the non-adaptive readout discriminability was 97 and 90% of that of the adaptive readout, for the 6 μm and 12 μm adaptation states, respectively (Figure 6E), indicating a markedly high level of generalisation of the optimal readout across different states of adaptation.

Noise correlations have been shown to depend on the stimulus features such as intensity-dependence in the somatosensory system (Adibi et al., 2013b) as well as in other sensory systems (Kohn and Smith, 2005; Oram, 2011; Middleton et al., 2012; Ponce-Alvarez et al., 2013). This suggests that instantaneous or short-term correlation structures may potentially provide an additional channel of information for sensory processing. The optimal linear readout scheme provides a framework for studying the effect of noise correlations on the efficiency of sensory processing in different adaptation conditions. Adibi et al. (2014) showed that neuronal adaptation increases signal (see Figure 5J) and noise correlations in population responses. By increasing signal and noise correlations, adaptation increases the redundancy of population responses. This adaptation-induced redundancy, can potentially limit the capacity of the cortical network to encode sensory information. The increased redundancy, in turn, may enhance the accuracy with which population responses represent sensory stimuli on trial-by-trial basis. The effect of noise correlation on information encoding/decoding depends on the direction of noise correlation (in the multi-dimensional space of joint population activity) relative to the direction of signal (Averbeck et al., 2006; Adibi et al., 2013b). In the non-adapted condition, noise correlations improve the accuracy of encoding/decoding for some populations and in some other populations, they were detrimental to population coding (Adibi et al., 2014). Similar opposing effects of noise correlation were observed in awake animals performing a texture discrimination task (Safaai et al., 2013). The differential effects of noise correlation can be attributed to the heterogeneity of neuronal populations; in a heterogeneous population, different neurons may exhibit a variety of signal directions in their responses relative to noise. This leads to opposing effects of noise correlation in the non-adapted state. In the adapted state, however, with decreased level of heterogeneity (Figure 5J), the population responses are more homogenised, showing less diversity in their direction of signal relative to noise. This leads to entirely detrimental effect of noise correlations in the adapted states (Adibi et al., 2014). Compatible with this scenario, it has been observed that noise correlations under sensory adaptation were always detrimental to information encoding/decoding (Adibi et al., 2014). The magnitude of the effect of noise correlations was greater in adapted states than non-adapted condition.

Based on these results, one might predict that ignoring noise correlations would be more detrimental to the performance of the readout under adaptation. On the contrary, ignoring noise correlations in the readout by taking into account only the diagonal elements of the pairwise neuronal response covariance matrix (denoted by subscript “diag” in Figure 6F) was less detrimental under adaptation compared to the non-adapted state. This discrepancy can be explained in terms of a greater increase in signal correlations relative to noise correlations under adaptation (Adibi et al., 2014).

## 3. Neuronal Mechanisms Underlying Sensory Adaptation in Somatosensory Cortex

A potential neuronal mechanism for adaptation is based on normalisation models (Heeger, 1991, 1992): the net activity of a population of neighbouring neurons increases the input conductance of the excitatory synapses, and hence results in the division of the activity of the neuron by the pool activity of the network, or shunting inhibition. Prolonged stimulation leads to a steady network activity and hence a stable input conductance. The key assumption then is that the changes of the synaptic conductance have a time constant; after prolonged stimulation, this reduced input conductance does not abruptly return to its initial state, but to a transient state for a few hundreds of milliseconds. This model is consistent with the electrophysiological findings in cat and monkey striate visual cortex and magnocellular cells in monkey LGN under contrast adaptation: a lateral shift in response function along the logarithmic contrast axis (Ohzawa et al., 1982, 1985; Sclar et al., 1989; Solomon et al., 2004), and has psychophysical correlates in human (Pestilli et al., 2007). However, the rightward shift along the stimulus amplitude axis (as in Figure 3D) is difficult to interpret in terms of a pure normalisation model. Moreover, the normalisation model cannot explain the decreased responsiveness along with the shifts illustrated in the response function of neurons in cat visual cortex and auditory nerve and inferior colliculus (Albrecht et al., 1984; Durant et al., 2007; Wen et al., 2009). An alternative mechanism is based on a tonic hyper-polarisation (Carandini et al., 1997) mainly due to decreased excitatory inputs (DeBruyn and Bonds, 1986; Vidyasagar, 1990; McLean and Palmer, 1996; Carandini et al., 1997), which is consistent with depression of synaptic excitation with repetitive electrical intracellular micro-stimulation of rat primary visual cortex neurons (Abbott et al., 1997). A general model consisting of these two mechanisms has been proposed for contrast adaptation in V1 neurons (Dhruv et al., 2011). Synaptic mechanisms, such as enhancement of inhibition (Dealy and Tolhurst, 1974) and depression of excitatory synapses (Finlayson and Cynader, 1995; Chance et al., 1998; Adorján et al., 1999; Carandini et al., 2002; Chung et al., 2002; Freeman et al., 2002; Wehr and Zador, 2005; Stevenson et al., 2010) have also been proposed as mechanisms underlying adaptation. *In vivo* experiments, however, demonstrated that suppression of inhibition by blockage of GABA_A_ receptors did not block sensory adaptation (DeBruyn and Bonds, 1986; Nelson, 1991).

In the rat whisker-mediated tactile system, based on current views of sensory adaptation, this phenomenon is mainly a result of short-term thalamocortical synaptic depression (Chung et al., 2002; Castro-Alamancos, 2004b; Khatri et al., 2004; Higley and Contreras, 2006; Heiss et al., 2008). Whisker-specific adaptation at the level of cortex (Katz et al., 2006) supports this mechanism. Assuming that tactile adaptation results mostly from short-term depression of thalamocortical synapses give rise to a number of predictions. One prediction is that increasing the intensity of stimulation, which is followed by higher presynaptic firing probability, results in greater depression during sustained sensory stimulation due to depletion of synaptic resources and the relatively slower recovery processes. This prediction, however, is in contrast to the observed intensity-dependent profile of sensory adaptation along the lemniscal pathway (Ganmor et al., 2010, also see Lampl and Katz, 2017). Ganmor et al. (2010) found that increasing the amplitude and velocity of whisker deflection does not increase the adaptation of synaptic responses in layer 4 neurons in the primary somatosensory cortex, but rather entailed less adaptation. In a series of electrophysiology recordings along the entire lemniscal pathway and first order ganglion neurons, this study showed that the source for this unexpected profile of adaptation—reduced degree of adaptation with increased intensity of stimulation—lies in PrV neurons of the trigeminal complex in the brainstem. Another body of literature, implicates adaptation of the thalamic spike timing (Wang et al., 2010b; Whitmire et al., 2016; Wright et al., 2021), suggesting that cortical adaptation is mainly a result of reduced bursting and adaptive changes of evoked synchronous spikes in the VPM. Recent studies indicate that the strength of synaptic connections between individual thalamic and cortical neurons is insufficient to evoke action potentials in cortical neurons. Instead, the cortex is driven by synchronous activity of thalamic populations (Bruno and Sakmann, 2006; Zucca et al., 2019). This suggests that the level of synchrony across thalamic neurons is a mechanism in regulating the flow of information to the cortex. Optogenetic elevation of the baseline activity in VPM is shown that does not adapt cortical neurons, and moderate level of sustained sensory stimulation has little effect on the response of cortical neurons to direct photo-stimulation of thalamocortical terminals in the cortex (Wright et al., 2021), suggesting little contribution of thalamocortical synaptic depression to sensory adaptation in the cortex in low and intermediate adaptation regimes. Further experiments are required to characterise the role of upstream structures such as thalamic sensory nuclei (Hartings et al., 2003; Khatri et al., 2004; Ganmor et al., 2010; Wang et al., 2010b), the laminar structure of the cortex (Allitt et al., 2017), intra-barrel and cross-barrel cortical circuitry (Katz et al., 2006) and the balance between excitatory and inhibitory connections (Higley and Contreras, 2006; Heiss et al., 2008; Malina et al., 2013) on sensory adaptation in the somatosensory system.

## 4. Adaptation and Coding Efficiency: An Information Theoretic Perspective

One of the current views of neuronal adaptation is that it is a mechanism by which neuronal responses adjust to the contextual changes in the environment in order to maintain the efficiency of neural codes. The efficiency can be quantified in relation to a given utility function (e.g., maximising discriminability or the rate of information) or a cost function (e.g., minimising the energy to transfer certain amount of information, or minimising the variance of estimation error).

### 4.1. Fisher Information

Fisher information (Fisher, 1922) is a well-known measure of coding accuracy that quantifies the amount of information that the neuronal responses carry about the sensory stimulus upon which the distribution of the neuronal responses depends. This measure has been used to characterise the effect of neuronal adaptation on the efficiency of coding in visual, auditory and somatosensory systems (Dean et al., 2005; Durant et al., 2007; Gutnisky and Dragoi, 2008; Adibi et al., 2013a). Adaptation can be considered as the procedure of matching the neuronal responses based on the distribution of the stimulus—a procedure known as ‘equalisation' (Laughlin, 1981; Nadal and Parga, 1994)—in order to maintain the efficiency (or optimality) of neuronal code. For the biological case where the neuronal response variance is stimulus dependent (Churchland et al., 2010; Adibi et al., 2013b), the optimality will be obtained when the square root of the Fisher information function is equal to the distribution of the stimulus. Thus, the peak of Fisher information function should be aligned with the most frequent stimulus in the environment. This is compatible with the Linsker's infomax principle (Linsker, 1988; van Hateren, 1992) and is equivalent to Barlow's redundancy reduction principle (Barlow, 1961, 2001; Atick, 1992; Redlich, 1993).

In vision, neuronal adaptation is shown to maintain the efficiency of the neuronal responses by scaling the response function of neurons with changes in the variance of input (Brenner et al., 2000), or by shifting the neuronal response functions with changes in the mean of input distribution. These adjustments have been reported in contrast adaptation in visual system (Ohzawa et al., 1982, 1985; Sclar et al., 1989; Solomon et al., 2004) and in sound level adaptation in auditory system (Dean et al., 2005) resulting in enhanced accuracy around the adapting (most frequent) stimulus (Dean et al., 2005; Durant et al., 2007). In somatosensory cortex, however, the shift in the coding accuracy to stimulus amplitudes above the adapting stimulus (Figure 3F) is not exactly consistent with the notion of equalisation or information maximisation. Equalisation predicts the peak of Fisher information aligns with the most frequent stimulus (here adapting stimulus). However, the Fisher information profile peaks at above adapting stimulus intensities (Figure 3F). As a result, neuronal adaptation filters out the most frequent features of the stimulus, and in turn, aligns the most sensitive portion of response curve (equivalent to peak of the Fisher information) to amplitudes above adapting stimulus. This potentially reflects a balance between homeostatic regulation of metabolic energy costs of spikes and maintenance of information content of neuronal responses, and in turn, tunes the network to the critical point of deviant detection. In the realm of whisker-mediated tactile world, for rodents, the most frequent stimuli may constitute the ambient noise in the surrounding environment, while behaviourally *significant* stimuli are those above the most frequent stimulus region. Adaptation can be considered as a mechanism to shift or scale the neuronal functions to maintain their accuracy for significant stimulus region by equalisation with respect to a “significance” function instead of the stimulus probability distribution function. In visual and auditory systems, however, adaptation tunes the neuronal responses to maintain the acuity for frequent stimuli. The more frequent stimuli, in these modalities, may be considered to constitute functionally or behaviourally significant stimuli.

### 4.2. Shannon Information

**Figure 7:**
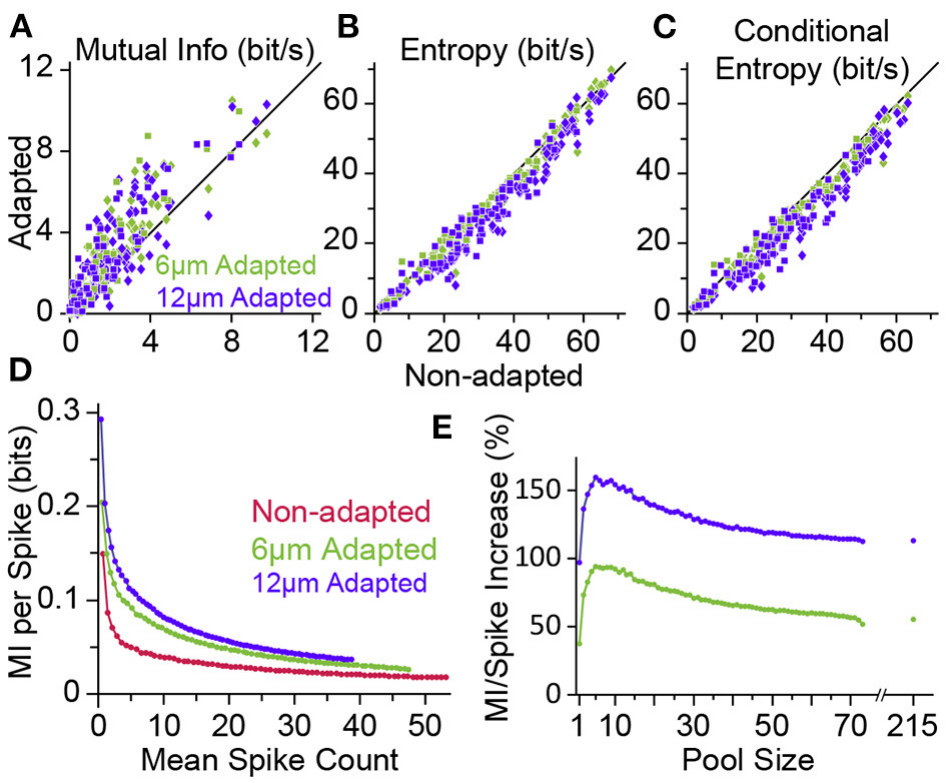
Adaptation enhances the information content of neuronal responses. **(A)** Mutual information (MI) between the whole stimulus set and the neuronal responses (from Figure 3) in adaptation condition (ordinate, 6 μm adaptation in green and 12 μm adaptation state in magenta) versus the non-adapted state (abscissa). Each data point corresponds to a single neuron (*n* = 73; square markers) or a cluster of multi-units (*n* = 86; diamonds). **(B)** As in **(A)**, but plotting the response entropy of individual neuronal recordings. **(C)** As in **(A,B)**, but plotting the response entropy conditional on stimulus. **(D)** Adaptation increases the average information content of individual spikes. Neuronal responses from different population sizes were pooled together and plotted against the average pooled spiking for every population size. **(E)** Percent increase in the single spike information in the adapted states relative to the non-adapted state. Data is from **(D)**. The data points after the break in the abscissa include multi-unit clusters with the single-unit data. From the firing rates, we estimate that the total population consisted of ~215 single units. Modified from Adibi et al. (2013a).

The transformation of physical attributes of the sensory environment into spiking activity is analogous to the concept of an “encoder” in the framework of coding theory in the realm of communications. In this framework, the neuronal computation is analogous to either that of the “source coding” or “channel coding.” An efficient source code is the one that maximises its entropy according to the distribution of sensory input. It is equivalent to minimising the redundancy in the neural code. Channel coding, on the other hand, adds patterns of redundancy in the transmitted signal to reduce the decoding error rate at the receiver over transmission through an erroneous channel. In an information theoretic framework, Adibi et al. (2013a,b) showed the amount of information in the neuronal responses about the amplitude of the sensory stimuli (in terms of the mutual information between the neuronal response and stimulus amplitude) increases with adaptation, hence enhancing coding efficiency (Figure 7A). This leads to the prediction that the sensory cortex may act as an adaptive entropy maximiser that increases the entropy of its codes (spike rate) (Attneave, 1954; Barlow, 1961; Srinivasan et al., 1982; Atick, 1992) similar to an optimum source coder in the realm of communications (Shannon, 1948). To identify the source of adaptation-induced enhancement in coding efficiency, Adibi et al. (2013a) decomposed the information content of neuronal responses (in terms of mutual information between neuronal responses and sensory stimuli) into its two fundamental components: the entropy of neuronal responses and the conditional response entropy given stimulus. Adaptation decreases the response entropy (Figure 7B) and the conditional response entropy (Figure 7C) at both the level of single neurons and the pooled activity of neuronal populations. The net effect of adaptation is to increase the mutual information between stimulus and neuronal responses. The information transmitted by a single spike also increases under adaptation, even when the overall rate of activity is matched across non-adapted and adaptation states (see Figure 7D).

The adaptation-induced increase in the information content of neuronal responses can be explained by a higher number of stimulus pairs for which adaptation increases Shannon information (and also discriminability) than the number of stimulus pairs (at amplitudes lower than adapting stimulus) of which adaptation reduces Shannon information (and discriminability) (Figure 6D, also see Adibi et al., 2013b). This lead to the net increase in the mutual information between neuronal responses and sensory stimuli and is consistent with the rightward shift in the Fisher information (Figure 3F).

## 5. Functional Roles of Sensory Adaptation

In light of the recent findings and studies on the phenomenology and physiology of sensory adaptation summarised in the previous sections, here, we present a number of (potential) functions of neuronal adaptation in the whisker-mediated somatosensory system, some of which proposed in other sensory systems as well.

### 5.1. Noise Reduction

Sensory adaptation desensitises the tactile sensory system during exposure to sustained or continuous stimulation. After some time, we tend to not notice ongoing sensory stimulation such as the scratching of a shirt on our body. At the perceptual level, the perceived intensity of tactile stimuli exponentially decreases over time during adaptation (Berglund and Berglund, 1970). The response of sensory neurons also exhibit similar exponential reduction trend at multiple stages of sensory processing (Hartings et al., 2003; Khatri et al., 2004; Musall et al., 2014; Allitt et al., 2017; Kheradpezhouh et al., 2017; Lampl and Katz, 2017). Consistently, as mentioned earlier, sensory stimuli with lower intensity than that of the adapting stimulus evoke little neuronal responses in the somatosensory cortex and thalamus (Adibi et al., 2013b, see also Figure 3). This reduced neuronal responsiveness to prevailing sensory stimuli, hence, provides a noise reduction mechanism to filter ambient stimuli at neuronal and cognitive levels.

### 5.2. Energy Conservation by Lowering Metabolic Costs

The fundamental basis of neural communication and brain function is through action potentials that neurons generate in order to transfer information to other neurons. The cost of a single action potential is high, with a net cost of ~2.4 × 10^9^ ATP molecules per action potential (Lennie, 2003). The fraction of energy consumption in neocortex associated with neural signalling is estimated to be 52% of total energy expenditure (Attwell and Laughlin, 2001; Lennie, 2003). This severely limits the number of action potentials that neuronal populations may persistently generate in response to sustained or repeated sensory stimuli in the environment. In the somatosensory cortex, adaptation improves neural coding efficiency at a reduced metabolic cost associated with spiking, due to a net decrease in neuronal responses (Adibi et al., 2013b, also see Figures 1B, 3D). Similarly, in the primary visual cortex, adaptation equalises population responses to stimulus orientations with different statistics, maintaining the overall rate of spiking averaged over time (Benucci et al., 2013).

### 5.3. Salience Processing and Deviance Detection

Survival in a dynamic and changing environment requires animals to detect unexpected sensory cues that signal necessary commodities (e.g., food and water), mates or danger from ambient sensory stimuli. For an urban rat searching for food in a dimly lit street, the tactile vibrations travelling through the asphalt from the movement of an approaching dog or car provide accurate estimate of the distance from the impending danger. Namib Desert golden moles process seismic sensory signals to detect prey (Narins et al., 1997). An animal, hence, should efficiently and quickly identify salient stimuli from the continuous stream of sensory signals in changing environments and react with appropriate behavioural responses. In tactile, behaviourally relevant events are potentially those with a higher intensity/acceleration compared with the prevailing sensory stimuli. This is particularly true for the generative mode of the whisker-mediated sensory system where interactions with textures and objects produce changes in the whisker trajectory beyond the baseline whisking action (Adibi, 2019). Sensory adaptation serves as a neuronal mechanism for salient stimulus detection by adjusting the sensitive region of the neuronal response functions to stimulus intensities above the background level (Adibi et al., 2013b; Musall et al., 2014, also see Figures 3 and **9**).

### 5.4. Efficient Neural Coding and Improved Discrimination Around Adapting Stimulus

In the natural environment, the prevailing diet of sensory stimuli varies over time. An efficient neural code adaptively matches the limited discriminative range of neuronal responses according to the distribution of sensory stimuli (Barlow and Földiák, 1989). This constitutes scaling the discriminative region of neuronal responses according to the variance of sensory stimuli (variance adaptation), as well as shifting the most discriminative point of the neuronal responses to the most frequent sensory stimulus (mean adaptation). Previous studies observed that variance adaptation maintains the information content of neuronal responses (Maravall et al., 2007, 2013). As for the mean adaptation, at the neuronal level, adaptation enhances discriminability of neuronal responses to stimuli around the adapting stimulus (Figure 6D, also see Adibi et al., 2014). We predict that the enhanced neuronal discrimination improves perceptual discrimination performances. Similar improved perceptual effects have been observed in humans for vibrotactile amplitude (Goble and Hollins, 1993) and frequency (Goble and Hollins, 1994) discrimination. In animal literature, improved performance was observed in rats performing a spatial whisker discrimination task (**Figures 10E–H**, also see Ollerenshaw et al., 2014). Future studies are required to investigate the effect of adaptation on perceptual discriminability.

### 5.5. Band-Pass Frequency Filtering Properties

During repetitive stimulation, adaptation leads to higher neuronal responsiveness at some frequencies than other frequencies, and therefore exhibiting frequency-filtering properties. Using different frequencies of whisker stimulation, Kheradpezhouh et al. (2017) observed that the net evoked response of over 90% of cortical neurons was at stimulation frequencies other than the maximum frequency (see also Figure 1). This constitutes a low- and band-pass frequency filtering property of sensory adaptation. However, thalamic neurons show more high-pass frequency response properties to sinusoidal stimulation at frequencies up to 40 Hz (Hartings et al., 2003). This could arise from lower spiking responses to sinusoidal stimuli at lower frequencies and hence at lower mean speed (Arabzadeh et al., 2003). A small yet significant subset of cortical neurons exhibit response facilitation (see Figure 1E) which is equivalent to high-pass filtering. Consistent with the diversity among cortical neurons in their frequency response profile (Allitt et al., 2017; Kheradpezhouh et al., 2017), at synaptic level, also diverse filtering properties have been identified across neurons (Anwar et al., 2017). During synaptic plasticity, facilitating synapses serve as high-pass filters as they are stronger at high pre-synaptic spiking frequencies, while depressing synapses serve as low-pass filters as they are stronger at low pre-synaptic spiking frequencies.

A misconception in the literature is to consider adaptation as a high-pass filter due to its slow adaptive process (for instance see Benda, 2021). Based on this view, high-frequency stimulus components that change on time scales faster than the adaptation processes are transmitted with a higher gain than lower frequency components. The flaw in this interpretation is that a slow filter in the time domain—which is characterised by a long impulse response—is assumed to be a low-pass filter in the frequency domain. However, there are no direct links between the time domain characterisation of a filter (slow or fast) and its frequency-domain characterisations (in terms of the passband frequency range of the filter). In fact, the frequency passband of a slow filter (with long impulse response function) and a fast filter (with short impulse response function) can be around any frequency. Hence, irrespective of the length of impulse response function (slow vs. fast), a filter may exhibit various low-pass, band-pass or high-pass properties.

### 5.6. Shift Between Integration and Coincidence Detection

Neurons conventionally are considered as coincidence detector or integrators depending on the time interval over which they accumulate and integrate input spikes (Abeles, 1982; König et al., 1996; Kisley and Gerstein, 1999). These modes of operation determine the way neural networks encode information; as rate coding scheme, or temporal coding scheme. Neuronal adaptation can be considered as a potential mechanism to shift the operating mode of the neuronal networks in a continuum between the two extreme modes of coincidence detector and temporal integrator. This can be implicated at the circuit level through different degrees of suppressive adaptation in either integrator or coincidence detector neurons within a neuronal population, or at single cell level through mechanisms of short term synaptic plasticity (D́ıaz-Quesada et al., 2014; Anwar et al., 2017) or through subthreshold adaptation modulation of the slope of the membrane potential (Kisley and Gerstein, 1999). Cortical layer 4 neurons including spiny stellate neurons which receive major thalamic input in barrel cortex have short integration intervals of a few milliseconds (Egger et al., 1999; Bruno, 2011; Adibi, 2019) and are driven by weak but synchronous thalamocortical input (Roy and Alloway, 2001; Bruno and Sakmann, 2006; Wang et al., 2010a,b). Varani et al. (2021) recently found that selective optogenetic inhibition of layer 4 neurons decreases sub-threshold responses to whisker deflections in the preferred direction of layer 2/3 neurons, while it increases responses to deflections in the non-preferred direction, leading to a change in the direction tuning. This allows a broader integration of signals in these neurons. During adaptation, thalamo-cortical synapses in layer 4 exhibit short-term depression (Chung et al., 2002; Lundstrom et al., 2010; D́ıaz-Quesada et al., 2014), potentially moving the operation point of cortical circuits from coincidence detection of thalamic inputs toward integration of inter-laminar and cortico-cortical inputs in layer 2/3 or infragranular neurons (for instance, see Jordan and Keller, 2020). Consistently, our unpublished data indicates that circumventing the sensory input to layer 4 neurons through direct optogenetic stimulation of layer 2/3 pyramidal neurons results in little adaptation in neuronal responses across cortical layers in the primary somatosensory cortex of mice.

### 5.7. Disambiguating Principal Features of Vibrotactile Sensation: Frequency and Amplitude

Previous electrophysiological studies revealed that neurons in the primary somatosensory cortex of rats encode vibrotactile stimuli in terms of the mean speed of whisker movement (Arabzadeh et al., 2003, 2004). The mean speed is equivalent to the product of the two fundamental features of vibrotactile stimuli: frequency and amplitude. While an increase in either feature increases the activity of cortical neurons, no measure of neuronal response (firing rates or temporal patterns) explicitly encodes one principal feature independently of another. This representation forms the basis of whisker-mediated tactile sensation in awake rats as well (Adibi et al., 2012). Consequently, two distinct stimuli with identical products of their frequency and amplitude are indistinguishable based on cortical neuronal responses as well as at the perceptual level. Stimulus dependent properties of neuronal adaptation (Ganmor et al., 2010; Adibi et al., 2013b; Mohar et al., 2013, 2015) potentially provides a neuronal mechanism to disambiguate encoding of the principal features of tactile stimuli from one another. Increases in the frequency and amplitude of stimulation has differential effects on the adaptation profile of neuronal responses. Thalamic and cortical neurons exhibit a higher level of response depression with increases in the frequency of stimulation (Kheradpezhouh et al., 2017). On the contrary, increased amplitude of stimulation results in lower level of adaptation (Ganmor et al., 2010; Mohar et al., 2013, 2015).

### 5.8. Parallel Processing of Stimulus Intensity

Adaptation alters the representation of external stimuli in a context-dependent manner, introducing response ambiguity. That is, an identical stimulus may evoke different responses depending on the context of stimulation, or conversely, different stimuli may evoke an identical response as context shifts. Under adaptation, PrV neurons better encode the fluctuations of the stimulus intensity when the intensity of stimuli is high, whereas neurons in the SpVi better encode weak tactile stimuli (Mohar et al., 2013, 2015). Together, the two nuclei provide and improve the overall coding of stimulus intensity at different stimulation intensity regimes (Mohar et al., 2015). The differential stimulus-dependent adaptation properties in the two parallel pathways of tactile system, the lemniscal and paralemniscal pathways, hence, may help in reducing the inherent ambiguity of neural coding of stimulus features in different adaptation conditions.

### 5.9. Adjusting Neuronal Receptive Fields

**Figure 8:**
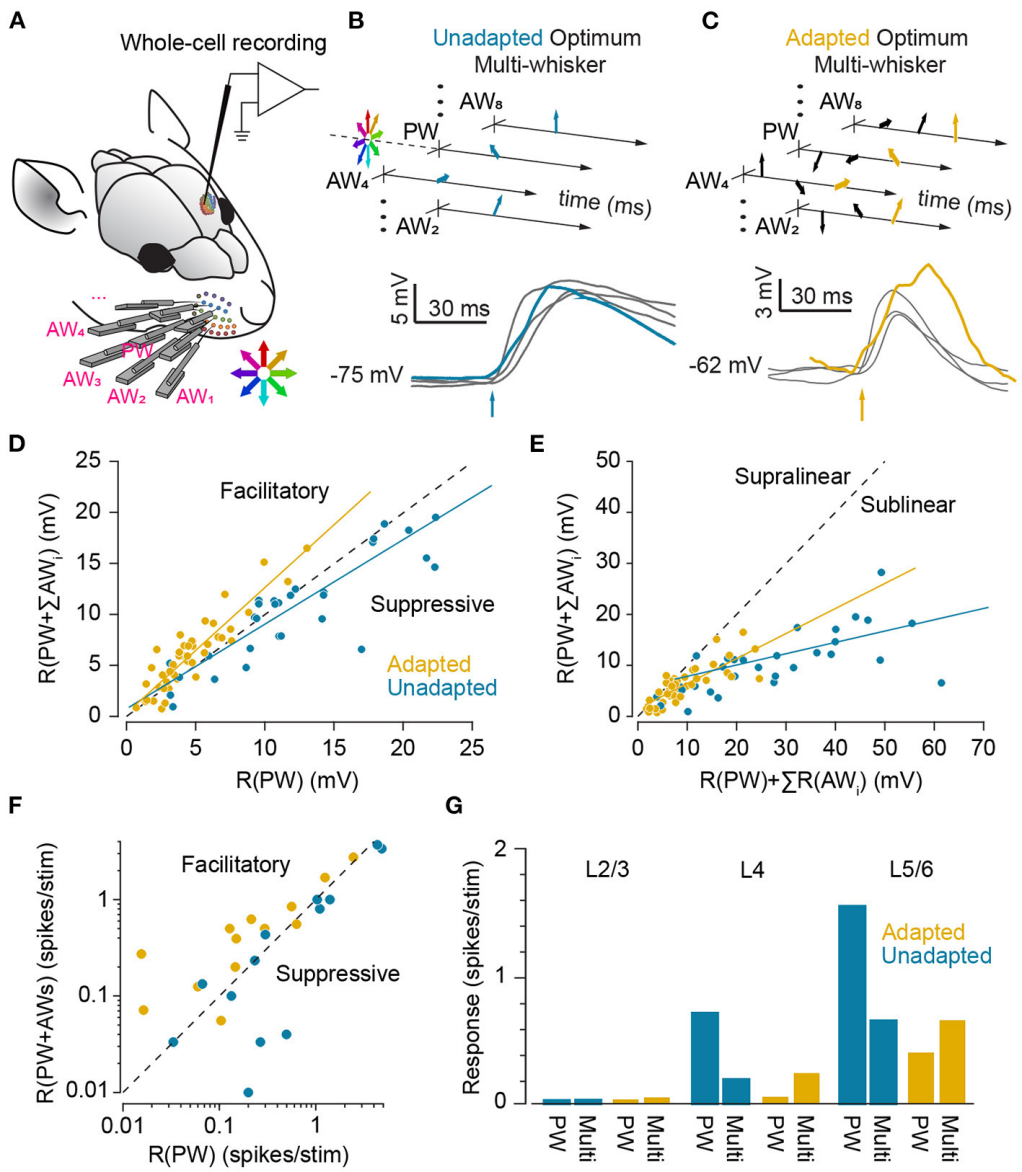
Adaptation enhances summation of synaptic inputs and allows surround stimuli to facilitate responses. **(A)** Cortical neurons in the vibrissal area of S1 were recorded intra-cellularly during complex multi-whisker stimulation to nine whiskers. The principal whisker (PW) and each of the eight adjacent whiskers (AW) were stimulated with high-velocity deflections of fixed temporal structure (5-ms rise and 5-ms decay) in arbitrary angles. Deflections occurred stochastically in time and direction at a frequency of ~9.1 Hz. The inset represents whisker directions in an eight angle-binned space. **(B)** The multi-whisker stimulus that evokes the maximum response for each neuron was determined and played back in isolation (unadapted). Trial-averaged post-synaptic responses of a neuron to each of the nine whiskers, R(PW) or R(AW) are shown in black. The arrow indicates stimulus onset. The response to the multi-whisker stimuli, R(PW + ΣAW_i_), is shown in cyan (unadapted). **(C)** As in **(B)**, but the multi-whisker stimulus was embedded within random surround stimuli (adapted, amber). **(D)** Response to the multi-whisker stimuli, R(PW + ΣAW_i_), is plotted against the responses to principal whisker deflection, R(PW). Surround inputs facilitated the PW response during adaptation by a factor of 1.28 ± 0.43 (*n* = 36, *p* < 10^−9^, sign test), but suppressed activity in unadapted neurons by a factor of 0.893 ± 0.269 (*n* = 33, *p* = 0.36, sign test). **(E)** Data from **(D)**, but plotting response to the multi-whisker stimuli, R(PW + ΣAW_i_), against the sum of responses to individual deflections, R(PW) + ΣR(AW_i_). Multi-whisker summation was closer to linear during adaptation (amber, slope = 0.491, *r* = 0.631, *p* < 10^−7^) compared to highly sublinear summation in unadapted condition (cyan, slope = 0.223, *r* = 0.442, *p* < 10^−9^). **(F)** As in **(D)**, but for the spiking activity of neurons (13 out of *n* = 33) that fired spikes in both conditions. Adaptation significantly facilitated spiking by a factor of 1.78 ± 1.04 (*p* = 0.02). In unadapted condition, responses were weakly suppressed or were not facilitated (0.85 ± 0.3, *p* = 0.30, sign test). **(G)** Same as **(F)**, but separated for layer 2 and 3 (L2/3), L4, and L5/6 neurons. The PW stimulation alone is the most effective driver of spiking activity in unadapted neurons in L4 and 5/6, but optimal multi-whisker stimuli were more effective under adaptation. Spiking activity in L2/3 remained sparse. Modified from Ramirez et al. (2014).

Majority of cortical neurons across different layers of whisker area of the somatosensory cortex exhibit multi-whisker receptive fields (Simons, 1978; Armstrong-James and Fox, 1987; Moore and Nelson, 1998; Ghazanfar and Nicolelis, 1999; Brecht and Sakmann, 2002; Brecht et al., 2003). Various spatial properties of receptive fields including the principal whisker, size, response latency and centre of mass in majority of cortical neurons exhibit stimulus-dependent changes (Le Cam et al., 2011). Furthermore, feature encoding properties of cortical neurons changes with the level of spatial correlation in multi-whisker sensory stimuli (Estebanez et al., 2012). Analogously, temporal multi-whisker stimulation patterns (see Figure 8) through whisker-specific adaptation mechanisms in cortical neurons (Katz et al., 2006; Ramirez et al., 2014) can potentially adjust the receptive field properties of neurons during different modes of behaviour in the environment; while sustained stimulation of principal whisker reduces the responsiveness to that specific whisker, this adaptation does not transfer to adjacent whiskers (Katz et al., 2006). This specificity, thus, maintains the responsiveness to stimulation of adjacent whiskers. Consequently, in the adapted state, stimulation of adjacent whiskers evokes a higher level of response relative to adapted responses to principal whisker stimulation. These results in a broader spatial extent of functional receptive field compared to the non-adapted state. Consistently, Ramirez et al. (2014) showed that while surround inputs in the non-adapted state are suppressive, as previously reported in the literature (Simons, 1985; Simons and Carvell, 1989; Brumberg et al., 1996), under adaptation, they are facilitatory and enhance the evoked responses to deflections of the corresponding principal whisker at the level of sub-threshold (Figures 8B–D) and spiking activity (Figure 8F). The adaptation-induced facilitation was stronger in layer 4, 5, and 6 neurons (Figure 8G). Conversely, adaptation by stimulation of all whiskers causes narrowing of the receptive fields (Katz et al., 2006; Ramirez et al., 2014). Adaptation also has been shown to affect multi-whisker integration of tactile stimuli (Figure 8E); integration of unadapted sub-threshold neuronal responses (in terms of PSPs) and spiking activity to preferred multi-whisker stimuli were highly sub-linear (Mirabella et al., 2001; Ramirez et al., 2014). However, when presented in a background of multi-whisker stimulation (adapted state), multi-whisker integration of responses were more linear (Ramirez et al., 2014). Consistently, Ego-Stengel et al. (2005) found that when principal and adjacent whiskers were simulated together at 0.5Hz, 59% of cortical neurons exhibit significant suppressive interactions, whereas response facilitation was found in only 6% of neurons. In contrast, at 8 Hz, a significant supra-linear summation was observed in 19% of the cells, with stronger effect along an arc compared to along a row. Such dynamic changes in the receptive fields could be a potential neuronal basis of invariant coding in whisker system. For instance, it might help to locate the position of whisker contact with respect to the head or the body instead of whiskers (for instance see Curtis and Kleinfeld, 2009). This position invariant information can potentially give rise to whisker-mediated coordination, and contribute to spatio-topic representations such as those in grid cells in the entorhinal cortex (Hafting et al., 2005) or head-direction cells in classic Papez circuit (Taube, 1998). Further experiments in awake and anaesthetised animals are required to understand adaptive changes in the multi-whisker receptive field of cortical neurons and their functional role. The adaptive adjustment of receptive fields forms the spatial dual of the adaptive shift between integration and coincidence detection modes in the time domain.

## 6. Link to Perception

Contextual modulations and adaptation are fundamental attributes of perceptual processing. Perceptual consequences of sensory adaptation have been commonly characterised in terms of repulsive after-effects. For instance, sustained exposure to lines at one orientation causes perceptual repulsion of the orientation of a subsequently viewed line, a phenomenon known as tilt after-effect (Gibson, 1933) with analogous repulsive effect observed in touch (Silver, 1969). Contextual effects of prolonged or repeated stimulation have been the subject of distinctly fewer studies in the tactile domain (Craig, 1993) while these effects are extensively studied in the visual system. The perceptual effects of sensory adaptation, and in particular whisker-mediated tactile system are still unknown, and limited to a few studies in the field. This is partly due to experimental challenges in training animals to withhold any action for the duration of adapting stimulus and further to isolate potential confounding effects of adapting stimuli on directly driving the behaviour. For a proportion of trials, animals may be distracted by the adapting stimulus. This, in turn, confounds measures of behaviour such as discrimination performance of the animals in a given sensory discrimination task. A solution to these challenges is to apply behavioural paradigms in which the adapting stimulus constitutes one of the stimuli based on which the animal makes perceptual judgements. For instance, in a discrimination task, one of the discriminanda could be the adapting stimulus. An example is a deviance or difference detection task in which the animals should detect a deviant stimulus in a repeated train of whisker deflections. The deviant stimulus could be a deviance in the location of the deflection (a different whisker), or in any physical feature of the stimulus including amplitude, duration or instantaneous frequency (inter-stimulus interval).

**Figure 9:**
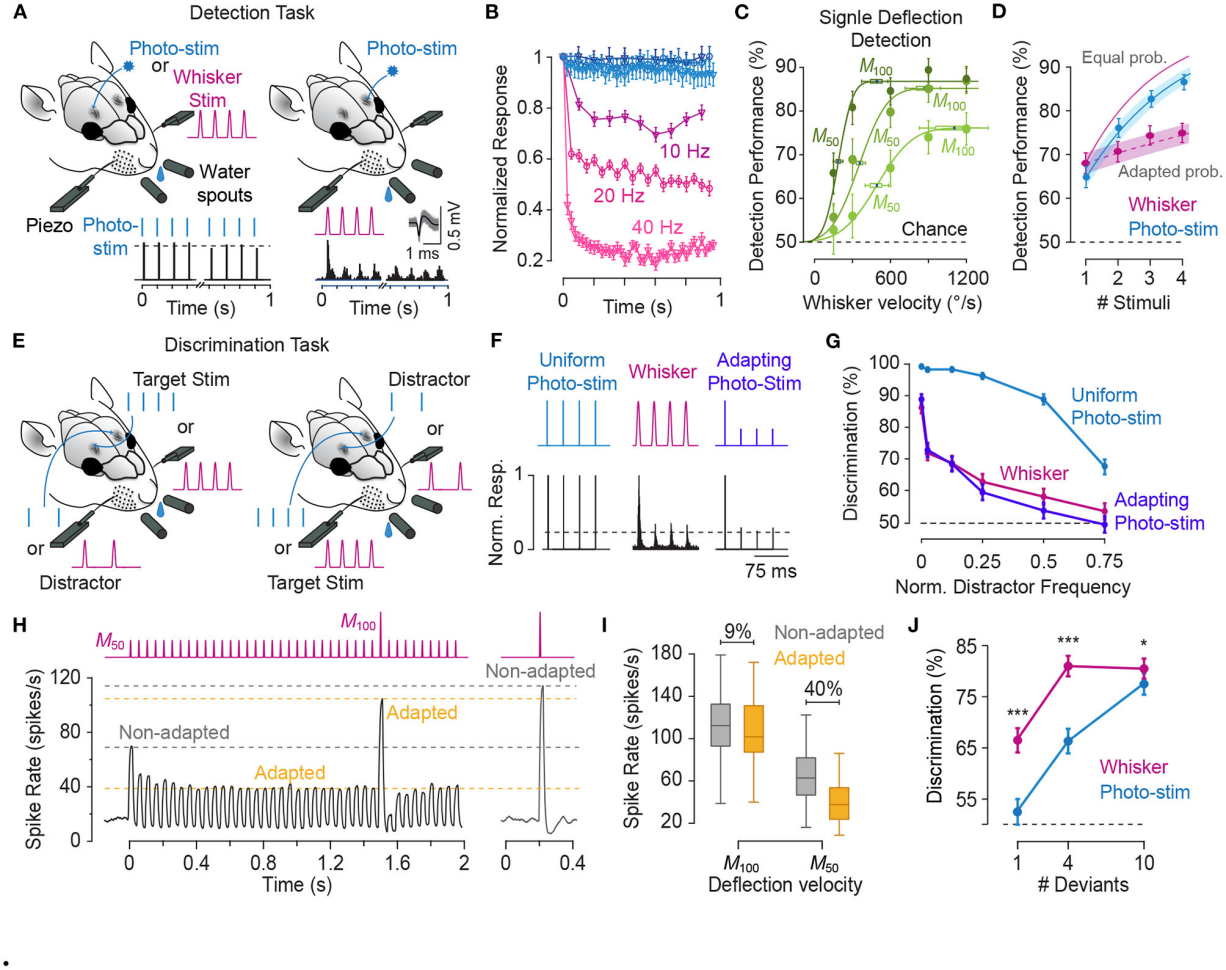
Circumventing cortical adaptation enhances detection and frequency discrimination, while adaptation improves deviant detection. **(A)** Detection task: in a 2-alternative choice task, rats were trained to detect stimulus that applied to either the left or the right C1 whisker or its barrel column. A reward was given if the animal responded correctly by licking at one of two water spouts on the side associated with the stimulus side. Whisker stimuli (red) consisted of individual or uniform sequences of pulses (single-cycle 120-Hz sine-wave). Photo-stimuli (blue) consisted of individual or sequence of 1-ms square-wave pulses. Insets show extracellular recording from two neurons to 40-Hz photo-stimulation (left) and stimulation of the principal whisker (right). PSTHs with spike rates normalised to the initial response. **(B)** Normalised response to whisker pulses (shades of red, 33 neurons) and photo-stimulation (shades of blue, 15 neurons) at 5, 10, 20, and 40 Hz frequencies (darker corresponds to higher frequencies) showing frequency-dependent adaptation to whisker stimulation and little adaptation to photo-stimulation. **(C)** Velocity-response curves for detection of single-pulse whisker deflections for 3 rats. M_50_ and M_100_ correspond to the turning point and the asymptote of the cumulative Gaussian function fitted to each curve, respectively. **(D)** Circles represent detection performances for sequences of 1–4 stimuli (with 25 ms inter-pulse interval) at M_50_. Detection of single whisker stimulus was 67.9%. However, detection rate increased by an average of 2.3 ± 0.93% for every additional stimulus in the sequence. This is lower than the prediction that every stimulus had an equal perceptual detectability (equal probability model, solid curves). When adaptation was considered by reducing the detection probability of subsequent pulses according to observed neuronal adaptation in **(B)** (adapted probability model, dashed line), the predicted curve matched the behavioural detection performance. In contrast with whisker stimulation, detection performance of direct cortical photo-stimulation (in blue) was well-explained by equal detection probability of individual pulses (solid blue curve). This indicates that non-adaptive neural activation (as in **B**) results in uniform perceptual weight of individual pulses in a sequence. **(E)** Frequency discrimination task; as in **(A)**, but the animals were trained to discriminate between a target stimulus (1-s long sequence of stimuli at 20 or 40 Hz) and a distractor with a lower frequency. **(F)** Three stimuli used in the discrimination task, with the corresponding normalised PSTHs (lower panels). Dashed line represents the adaptation level to whisker stimulus at 40 Hz. The whisker stimuli and uniform photo-stimulation pulses were set at M_100_ level. for the adapting photo-stimulation, the irradiation level of the initial pulse was set to M_100_, while the irradiation of subsequent pulses was reduced to that matching adaptation to whisker stimulation. **(G)** Frequency discrimination performances plotted against the frequency of distractor normalised to that of the target stimuli. Comparing discrimination performances for adaptation-free uniform photo-stimulation (blue) to whisker stimulation (red) reveals that adaptation reduces frequency discrimination performances. Adapting photo-stimulation (magenta) mimics whisker stimulation, resulting in reduced frequency discrimination performances. **(H)** Adaptation facilitates detection of deviant stimuli. The black trace shows average neural responses (*n* = 33) to a 2-s long 20-Hz whisker stimulation sequence (at the mean M_50_ velocity of 350°/s) with a single deviant (at M_100_, 850°/s). Response amplitude to subsequent pulses was decreased by 40% relative to the initial pulse, whereas deviant response amplitude remained close to non-adapted single-pulse response. **(I)** As in **(H)**, but using whisker-box plot. The box shows the first and third quartiles, the inner line is the median. Box whiskers represent minimum and maximum values. **(J)** Deviant stimulus detection performance as a function of number of deviant stimuli, was higher for whisker stimuli than photo-stimulation. Deviant detection task: two base sequences of either whisker or photo stimuli (at M_50_ amplitude, 20-Hz frequency and duration of 2 s) presented bilaterally. The target sequence (left or right) contained 1, 4, or 10 deviant pulses of M_100_ in amplitude at a random time after 1.5 s. Rats were rewarded upon successful identification of the deviant-containing target sequence. Error bars indicate s.e.m. **(B)** and 95% CI (elsewhere). Modified from Musall et al. (2014).

Using a similar behavioural approach, Musall et al. (2014) found rats performing a detection (Figure 9A) or frequency discrimination task (Figure 9E) exhibited reduced performance to detect or discriminate sequence of peripheral whisker stimulations compared to when the neuronal responses to subsequent stimuli were not adapted—using intensity-matched photo-stimulation, see Figures 9B,C—as shown in Figures 9D,G. Conversely, an optogenetic pattern of stimulation that mimicked sensory adaptation in cortical neurons replicated the whisker stimulation discrimination and detection performances (Figures 9F,G). Adaptation, however, enhanced the accuracy of rats detecting deviant whisker stimuli embedded within a series of whisker deflections at a lower amplitude (Figures 9H–J). This finding reveals that adaptation enhances perception of deviant stimuli with higher amplitudes than the prevailing stimuli while reducing the acuity under steady states of adaptation.

**Figure 10:**
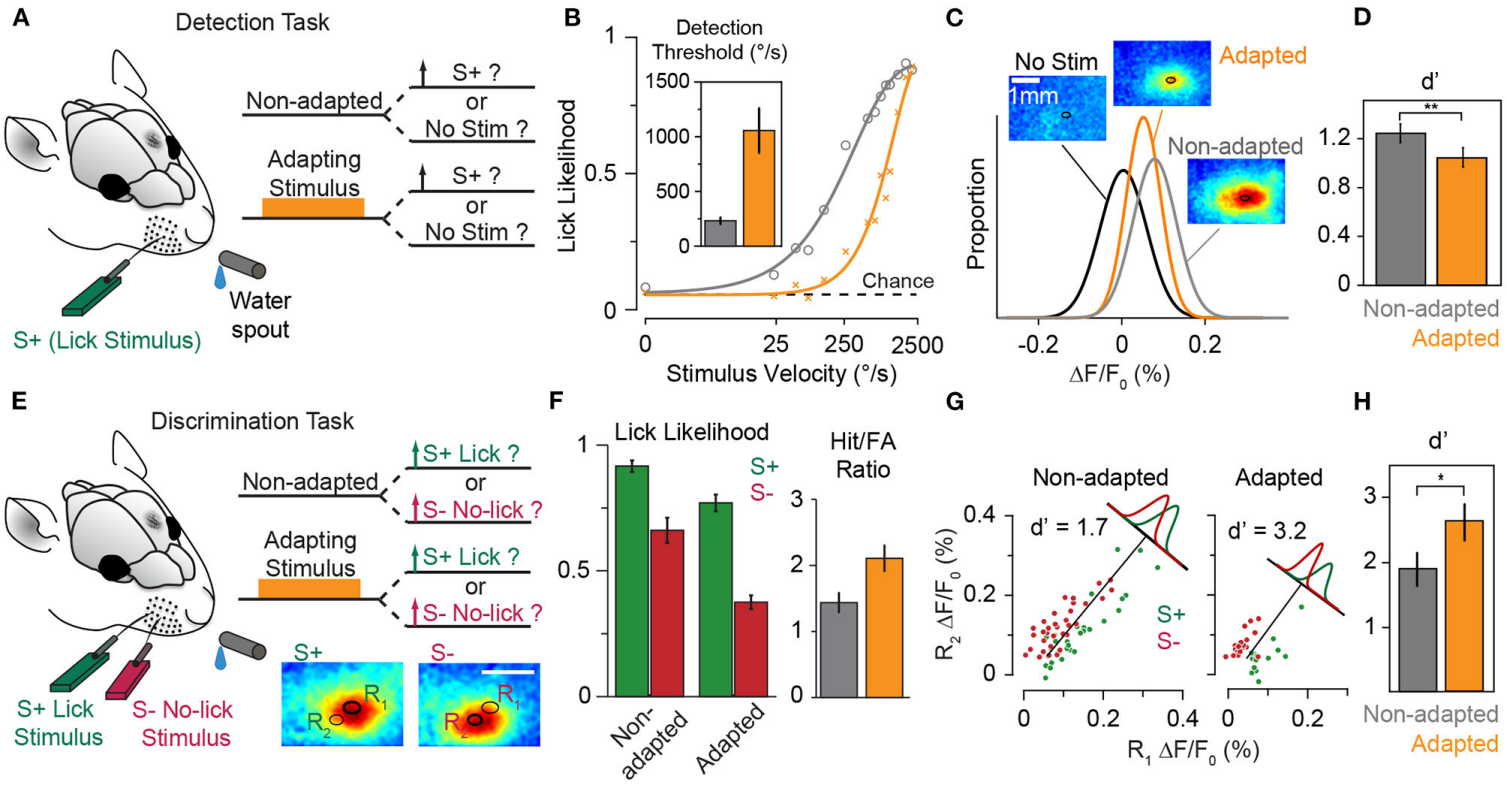
Adaptation decreases stimulus detectability while improves discriminability. **(A)** Detection task: a piezoelectric actuator was placed on a single whisker, and a variable velocity probe stimulus (S+) was presented at a randomised time. The probe was preceded by an adapting stimulus on 50% of trials. Animals had a 1 s window following the stimulus in which to emit a lick response to receive a water reward. **(B)** Psychometric curve—response likelihood as a function of stimulus—averaged across all animals, for the non-adapted (grey) and adapted (amber) conditions. The black dashed line indicates the chance performance level (licks in catch trials). The inset depicts that behavioural detection thresholds are increased with adaptation. Each bar represents the perceptual detection threshold, measured as the 50% point of the sigmoidal fit (M_50_). Error bars represent s.e.m. **(C)** Ideal observer of neuronal activity. Neural activity was measured using voltage-sensitive dye (VSD) imaging of cortex within an approximately barrel-sized (300–500 μm in diameter) region of interest (ROI) time-averaged over 10–25 ms after stimulus onset. The ROI was defined as the 98% height contour of the 2D Gaussian fit to the trial-averaged non-adapted responses. The insets show the corresponding trial-averaged VSD images for no-stimulus (pre-stimulus, in black), non-adapted (grey), and adapted (amber) with the ROIs outlined in black. An average fluorescence within the ROI was extracted from each trial for ideal observer analysis. **(D)** The stimulus detectability of ideal observer decreased with adaptation. The d' value, a measure of the separation of stimulus vs. no-stimulus distributions, decreased with adaptation (*p* <0.005, *n* = 18, paired *t*-test). Error bars represent s.e.m. **(E)** Discrimination task: two piezoelectric actuators were used to stimulate two nearby whiskers. On a given trial either the whisker associated with the S+ (lick stimulus) or the nearby whisker associated with the S- (no-lick stimulus) was deflected with equal probability. Whisker deflection was at a fixed supra-threshold velocity. Animals were rewarded for responses to the S+ stimulus (hit), but were penalised with a time-out for responses to deflections of the S- whisker (false alarm, or FA). The insets depict neuronal responses to the two whisker stimuli (S+ and S-). Two responses were calculated for each single trial: the average fluorescence within the principal barrel area (bold ellipse), and that within the adjacent barrel area (thin ellipse) using the same method as in **(C)**. The white scale bar in the inset represents 1 mm. **(F)** Adaptation improves the behavioural discriminability characterised in terms of the ratio of the proportion of hit trials to FA trials. **(G)** Example of linear discriminant analysis of neuronal responses to S+ and S- in the non-adapted (left panel) and adapted (right panel) conditions. Each data point corresponds to a single trial with the response from ROI associated with S+ principal whisker (ordinate) vs. the response from the ROI associated with S- principal whisker (abscissa). Neuronal response distributions to S+ and S- were obtained by projection of data points onto the axis orthogonal to the best discriminant line. The d' separation measure was then calculated for the two probability distributions. The d' values in this example were 1.7 (non-adapted) and 3.2 (adapted). **(H)** Adaptation enhances discrimination performance of the ideal observer of neuronal activity (*p* <0.05, *n* = 9, paired *t*-test). Error bars represent s.e.m. Modified from Ollerenshaw et al. (2014).

In another study, using a combination of single-whisker detection task and a two-whisker spatial discrimination behavioural task (Figures 10A,E), Ollerenshaw et al. (2014) showed that sensory adaptation improves spatial discriminability of stimulation of either whisker in behaving animals at the expense of reduced detectability of whisker stimulation (Figure 10B vs. Figure 10F). These results are consistent with the performance of an ideal observer of neuronal activity from voltage sensitive dye (VSD) imaging of the somatosensory cortex in anaesthetised rats (Figure 10, Ollerenshaw et al., 2014); the non-adapted neuronal responses to deflection of each whisker were spatially wide-spread with significant spatial overlap (Figure 10C and inset in Figure 10E), while the adapted responses were spatially constrained with decreased spatial overlap (Figures 10C,G, also see Zheng et al., 2015). Accordingly, similar to behavioural results, while adaptation decreases the detection performance of the ideal observer of neuronal responses (Figure 10D), it enhances its discrimination performance (Figure 10H). These findings demonstrate a trade-off between detectability (detecting the presence or absence of stimulus) and spatial discriminability (distinguishing stimulus location) up to a moderate level of adaptation which is compatible with the frequency range of natural whisking. For higher levels of adaptation, however, suppression of neuronal responses causes decreased detectability and discriminability.

In contrast to vibration sensation (Hill, 2001), evidence that wild rodents use their vibrissae to distinguish tactile textures including roughness of surfaces in the natural world is yet to be found. In the laboratory environment, when trained, rats and mice are capable of distinguishing textures such as rough vs. smooth surfaces using their micro- and macro-vibrissae (Carvell and Simons, 1990; von Heimendahl et al., 2007), even by a single whisker (Park et al., 2020). The accuracy with which rats and mice distinguish textures is comparable to that of primate fingertips (Carvell and Simons, 1990). Tactile texture sensation requires the active mode of sensation when an animal's whiskers palpate an object/texture during exploratory whisking. The temporal profile of whisker stick-slip events is hypothetised to determine signatures of tactile textures (Arabzadeh et al., 2005) and forms the basis of whisker-mediated texture perception (Wolfe et al., 2008; Isett et al., 2018). High-speed whisker tracking during texture discrimination (Zuo and Diamond, 2019) revealed texture-informative whisker kinetics could be represented by three features respectively related to shape, motion, and angle of whisker during contact. These kinematic features account for the amount of evidence in each whisker touch and correlate with neuronal activity in the primary and secondary somatosensory cortices (Zuo and Diamond, 2019). Interestingly, an exponentially-decreasing weighted integration of sequential touches fits well the behavioural choices compared to a uniform integration or a recency model in which the most recent touch is weighted more. A similar exponentially-decreasing weighted integration of neuronal activity of the primary and secondary somatosensory cortical neurons with similar time constant as that of the whisker kinematic features accounts for behaviour (Zuo and Diamond, 2019). The exponentially-decreasing form of integration could potentially represent the perceptual weight of neuronal activity as they adapt and shape perception. As S1 neurons progressively adapt to the sequence of whisker contacts (Allitt et al., 2017), the amount of information/evidence in their responses decreases with adaptation over time, and hence, they contribute less to the perception. This is consistent with exponentially-decreased perceived intensity of repeated tactile stimuli in human subjects (Berglund and Berglund, 1970) and in rats (Musall et al., 2014). Further experiments are required to understand the perceptual and neuronal effects of adaptation during the actively whisking mode of tactile sensation.

## Author Contributions

MA drafted the manuscript. Both authors edited the manuscript and approved the final version.

## Funding

This work is supported by the University of Padua under the 2019 STARS Grants programme (CONTEXT, Context matters: from sensory processing to decision making) to MA. MA was supported by an Australian Research Council DECRA fellowship (DE200101468) and CJ Martin Early Career Fellowship (GNT1110421) from the Australian National Health and Medical Research Council (NHMRC). IL was supported by DFG (SFB 1089), Human Frontier Science Program Grant, Israel Science Foundation (ISF 1539/17), BSF Grant 2019251, and the Marianne Manoville Beck Laboratory for Research in Neurobiology in Honor of her Parents Elisabeth and Miksa Manoville.

## Conflict of Interest

The authors declare that the research was conducted in the absence of any commercial or financial relationships that could be construed as a potential conflict of interest.

## Publisher's Note

All claims expressed in this article are solely those of the authors and do not necessarily represent those of their affiliated organizations, or those of the publisher, the editors and the reviewers. Any product that may be evaluated in this article, or claim that may be made by its manufacturer, is not guaranteed or endorsed by the publisher.
